# Supplementary material for: Assessment of the implementation fidelity of a strategy to scale up integrated care in five European regions: a multimethod study
Source: BMJ Open. 2020 Mar 18;10(3):e035002. doi: 10.1136/bmjopen-2019-035002 (PMC7150600; doi:10.1136/bmjopen-2019-035002)
Supplement: Supplementary data [file bmjopen-2019-035002supp002.pdf]

Appendix B Details of the interviews

**Participants**	**Main theme discussed**	**Time of interview**
Project members responsible for the coordination of the project (2 members)	Views and experiences in undertaking the work regarding the coordination of the project.	August 2018
Project members responsible for implementing the first step of the strategy (3 members)	Views and experiences in undertaking the work regarding identification of maturity requirements of selected local IC interventions (GPs) which have the potential for scaling up	First interview in June 2017; second interview on revised methodology September 2018
Project members responsible for the developments of the tool within the strategy (2 members)	Views and experiences in undertaking the work on the refinement of MM	August 2018
Project members responsible for the implementation of the second step in the strategy (2 members)	Views and experiences in undertaking the work regarding the self-assessment process of SCIROCCO regions	July 2018
Project members responsible for the implementation of the third step in the strategy (2 members)	Views and experiences in undertaking the work regarding coaching and twinning of regions	July 2018
Project members responsible for the collecting lessons learned on using the tool and the process of scaling up (1 member, and 1 member provided feedback to the summary of interview)	Views and experiences in undertaking the work regarding the collection lessons learned on the process of scaling up	July 2018
