# Supplementary material for: Assessment of the implementation fidelity of a strategy to scale up integrated care in five European regions: a multimethod study
Source: BMJ Open. 2020 Mar 18;10(3):e035002. doi: 10.1136/bmjopen-2019-035002 (PMC7150600; doi:10.1136/bmjopen-2019-035002)
Supplement: Supplementary data [file bmjopen-2019-035002supp003.pdf]

Appendix C Timetable planning project and executed activities

Blue: anticipated duration of task. Orange: actual duration of task spent during implementation as indicated in reports. Yellow: deviated actual duration spent as indicated by other reports.

	1									2												3											4
Month	Apr 16 (M1)	May 16 (M2)	June 16 (M3)	July 16 (M4)	August 16(M5)	Sep 16 (M6)	Oct 16 (M7)	Nov 16 (M8)	Dec 16 (M9)	Jan 17 (M10)	Feb 17 (M11)	Mar 17 (M12)	Apr 17 (M13)	May 17 (M14)	June 17 (M15)	July 17 (M16)	Aug 17 (M17)	Sep 17 (M18)	Oct 17 (M19)	Nov 17 (M20)	Dec 17 (M21)	Jan 18 (M22)	Feb 18(M23)	Mar 18 (M24)	Apr 18 (M25)	May 18 (M26)	June 18 (M27)	July 18 (M28)	Aug 18 (M29)	Sep 18 (M30)	Oct 18 (M31)	Nov 18 (M32)	
Step 1 Maturity requirements in GPs in five regions																																	
Viability assessment of GP																																	
Data collection																																	
Maturity requirements																																	
							1st																										
																							2nd										
Step 2 Self-assessment process in five regions																																	
Self-assessment process in five European regions																																	
Strengths and weaknesses of the European regions in integrated care																																	

Step 3 Twinning and coaching activities in five regions																																	
Development of methodology for twinning and coaching																																	
Coaching and twinning acitvities																																	
Development of Action Plans																																	
