# Supplementary material for: Assessment of the implementation fidelity of a strategy to scale up integrated care in five European regions: a multimethod study
Source: BMJ Open. 2020 Mar 18;10(3):e035002. doi: 10.1136/bmjopen-2019-035002 (PMC7150600; doi:10.1136/bmjopen-2019-035002)
Supplement: Author's manuscript [file bmjopen-2019-035002.draft_revisions.pdf]

BMJ Open

BMJ Open is committed to open peer review. As part of this commitment we make the peer review history of every article we publish publicly available.

When an article is published we post the peer reviewers' comments and the authors' responses online. We also post the versions of the paper that were used during peer review. These are the versions that the peer review comments apply to.

The versions of the paper that follow are the versions that were submitted during the peer review process. They are not the versions of record or the final published versions. They should not be cited or distributed as the published version of this manuscript.

BMJ Open is an open access journal and the full, final, typeset and author-corrected version of record of the manuscript is available on our site with no access controls, subscription charges or pay-per-view fees (http://bmjopen.bmj.com).

If you have any questions on BMJ Open's open peer review process please email info.bmjopen@bmj.com

BMJ Open

Implementation fidelity study of a strategy to scale-up integrated care in Europe

Journal:	*BMJ Open*
Manuscript ID	bmjopen-2019-035002
Article Type:	Original research
Date Submitted by the Author:	15-Oct-2019
Complete List of Authors:	Grooten, Liset; Vrije Universiteit Brussel, Family Medicine and Chronic Care<br>Fabbricotti, Isabelle; Erasmus University Rotterdam, Erasmus School of Health Policy & Management<br>Vrijhoef, Hubertus; Maastricht Universitair Medisch Centrum+, Patient & Care; Panaxea<br>Devroey, Dirk; Vrije Universiteit Brussel, Family Medicine and Chronic Care
Keywords:	Organisation of health services < HEALTH SERVICES ADMINISTRATION & MANAGEMENT, QUALITATIVE RESEARCH, Organisational development < HEALTH SERVICES ADMINISTRATION & MANAGEMENT, Change management < HEALTH SERVICES ADMINISTRATION & MANAGEMENT

SCHOLARONE™
Manuscripts

I, the Submitting Author has the right to grant and does grant on behalf of all authors of the Work (as defined in the below author licence), an exclusive licence and/or a non-exclusive licence for contributions from authors who are: i) UK Crown employees; ii) where BMJ has agreed a CC-BY licence shall apply, and/or iii) in accordance with the terms applicable for US Federal Government officers or employees acting as part of their official duties; on a worldwide, perpetual, irrevocable, royalty-free basis to BMJ Publishing Group Ltd ("BMJ") its licensees and where the relevant Journal is co-owned by BMJ to the co-owners of the Journal, to publish the Work in this journal and any other BMJ products and to exploit all rights, as set out in our licence.

The Submitting Author accepts and understands that any supply made under these terms is made by BMJ to the Submitting Author unless you are acting as an employee on behalf of your employer or a postgraduate student of an affiliated institution which is paying any applicable article publishing charge ("APC") for Open Access articles. Where the Submitting Author wishes to make the Work available on an Open Access basis (and intends to pay the relevant APC), the terms of reuse of such Open Access shall be governed by a Creative Commons licence – details of these licences and which Creative Commons licence will apply to this Work are set out in our licence referred to above.

Other than as permitted in any relevant BMJ Author's Self Archiving Policies, I confirm this Work has not been accepted for publication elsewhere, is not being considered for publication elsewhere and does not duplicate material already published. I confirm all authors consent to publication of this Work and authorise the granting of this licence.

For peer review only

Implementation fidelity study of a strategy to scale-up integrated care in Europe

Liset Grooten, MSc, Department of Family Medicine and Chronic Care, Vrije Universiteit Brussel, Brussels, Belgium (fennechien.grooten@vub.be, corresponding author)

Isabelle Natalina Fabbricotti, PhD, Erasmus School of Health Policy & Management, Erasmus University Rotterdam, (fabbricotti@eshpm.eur.nl)

Dirk Devroey, PhD, Department of Family Medicine and Chronic Care, Vrije Universiteit Brussel, Brussels, Belgium (Dirk.Devroey@vub.be)

Hubertus Johannes Maria Vrijhoef, PhD, Department Patient & Care, Maastricht University Medical Center, Maastricht, the Netherlands; Panaxea B.V., Amsterdam, the Netherlands; Department of Family Medicine and Chronic Care, Vrije Universiteit Brussel, Brussel, Belgium, Brussels, Belgium (bert.vrijhoef@panaxea.eu)

Corresponding author

Liset Grooten, MSC

Department of Family Medicine and Chronic Care

Faculty of Medicine and Pharmacy

Vrije Universiteit Brussel

P.O. 103, B-1090 Brussels, Belgium

Tel.: + 32 2 477.43.11

Fax.: + 32 2 477.43.01

Fennechien.grooten@vub.be

For peer review only

Abstract

Objective The SCaling IntegRated Care in COntext (SCIROCCO) project tested a step-based scaling up strategy to explore what and how to scale-up integrated care initiatives in five European regions. To gain a profound understanding of which factors influence the implementation of this strategy, the objective of this study was to assess the extent to which the SCIROCCO strategy was implemented as planned.

Design Multi-method qualitative study

Methods The extended version of the conceptual framework for implementation fidelity was used to evaluate what mechanism and factors influence the implementation of the scaling up strategy. Data were collected in the five participating European regions during the intervention period. Data collection methods included: key informant interviews, focus groups, questionnaire studies, and project documents.

Results All three main steps of the scaling up strategy were implemented with acceptable fidelity. Variations were observed in the duration of implementing the steps in the regions. Also, variations were observed in the coverage of experts to participate in the steps of the strategy. Several factors were observed to influence the implementation: facilitation conditions (i.e. good coordination for implementation) and participant responsiveness (i.e. a positive experience of participants in the organised study visits). Factors which may have moderated adherence to the original plan of the strategy were found in facilitating conditions (i.e. in the flexible approach), participant recruitment factors (i.e. adaptions of the procedure by the regions) and contextual factors (i.e. the level of development of integrated care).

Conclusion This was the first study to assess implementation fidelity of a European project which used a step-based scaling up strategy in five European regions. Similar European projects that are based on collaboration between several European regions can learn from the lessons captured in SCIROCCO and can become more aware of the facilitating factors and pitfalls of implementing such projects.

Key words: Implementation fidelity, integrated care, scaling up

Strengths and limitations of this study:

- This study was the first to assess the extent to which a step-based scaling up strategy in five European regions was implemented as planned within a European project.
- All five components of the scaling up strategy were implemented with acceptable fidelity. The insights obtained could support other regions interested to use the SCIROCCO tool and processes for achieving knowledge transfer and ultimately scale-up their integrated care initiatives.
- Direct involvement as researchers in this EU project provided a unique opportunity to follow the project closely, and to obtain solid cooperation to conduct the research activities. However, choices needed to be made regarding the data collection to align the research activities within the larger EU project and to be feasible to undertake it within the project time.

Background

For peer review only - http://bmjopen.bmj.com/site/about/guidelines.xhtml

Numerous integrated care initiatives have been developed around the world to bring about accessible, high quality, effective and sustainable health and social care [1]. Consequently, valuable lessons on the design and implementation of the transformation towards integrated care have been gained [2]. However, less known is what factors contribute to the progress and success of integrated care (IC) initiatives. Obtaining such insights is challenging, which is partially induced by deficient and absent evaluation and measurement of IC initiatives, resulting in the lack of evidence on the working mechanisms in IC [3]. In addition, many of the available assessment tools for IC have been poorly or insufficiently validated [4]. As a result, there is a lack of insight in how, when and why integrated care initiatives achieve certain results [5,6]. This lack of knowledge means that it is hard to learn from experiences [7–9] and to know to what extent and in which form integrated care initiatives can be implemented in different settings [10,11].

To explore how to learn from experiences and knowledge of current integrated care initiatives, the Scaling IntegRated Care in Context (SCIROCCO) project tested a structured approach intended to facilitate upscaling of successful initiatives. This EU subsidised project did so by focusing on the context and environment (i.e. the regional delivery system and political and organisational environment) of IC initiatives. A step-based scaling up strategy was implemented as part of the project with the aim to facilitate the implementation of Good Practices (GPs) in IC at local, regional or country level by recognising the maturity requirements of GPs and health and care systems in order to achieve scaling up and knowledge transfer amongst European Member States [12]. The strategy was implemented in five regions (Basque Country (Spain), Norrbotten (Sweden), Puglia (Italy), Olomouc (Czech Republic) dan Scotland (United Kingdom).

The SCIROCCO step-based strategy consists of multiple components which are planned to be implemented in five different settings. The implementation of complex and multi-component interventions rarely happens as planned [13]. A variety of factors can influence the implementation of a strategy (like SCIROCCO's), such as the context in which an intervention is implemented [14] and the complexity of the intervention [15]. The local contexts of the different regions in SCIROCCO might have led to necessary adaptations during the implementation of the scaling up strategy and these adaptations might influence the outputs of the strategy. One way to evaluate the implementation of the strategy is by exploring the implementation fidelity. Implementation fidelity refers to "the degree to which…programs are implemented…as intended by the program developers."[13] This study, therefore, assessed the fidelity of the implementation of the step-based strategy to examine how the SCIROCCO strategy was delivered in practice.

The approach we used for the assessment of implementation fidelity, is based on the work of Carroll et al. [16], and Hasson [17]. The measurement of implementation fidelity is a measurement of adherence, with its subcategories content, frequency, duration and coverage (dose) [16]. Several moderating factors are suggested in the framework to influence the level of fidelity. These factors are: participant responsiveness, program complexity, comprehensiveness of policy description, strategies to facilitate implementation, quality of delivery, recruitment, and context. In this paper, we present findings about the extent to which ''the activities within the SCIROCCO project have been implemented in line with expectations and if, how, and how far relevant initiatives have been developed between 2016 and 2018'' [18]. In this way, factors which facilitated the implementation and factors which were not conducive to the implementation of the strategy are revealed. These insights in the implementation and contextual conditions of the SCIROCCO strategy will provide lessons for those interested in SCIROCCO's step-based approach and future use of its tool and processes to reach progress in the integration of care services. In addition, we aim to contribute to a better understanding on what works in integrated care initiatives and how successful ones can be scaled up.

Methods

SCIROCCO's scaling up strategy

For peer review only - http://bmjopen.bmj.com/site/about/guidelines.xhtml

The SCIROCCO consortium was responsible for the implementation of the strategy. The implementation of the step-based scaling up strategy in the five participating regions was the specific responsibility of the regional partners in five regions. Three regional partners were lead partners for the development of one of the three steps of the scaling up strategy (in collaboration with the other partners within the project). The other partners (referred to as supportive partners) were responsible for the supportive activities within the wider project, such as project coordination, dissemination activities, development of the tool, capturing lessons on the strategy and one independent evaluator (more details are provided in our study protocol). The plan and details on how to implement the step-based strategy were described in the Grant Agreement (protocol). However, not all exact details have been provided as it was indicated that the exact outline and content of several activities were to be developed during implementation. The description of the step-based strategy, as provided in the protocol, is briefly described below.

Step 1: Assessment of maturity requirements in selected GPs

The first step of the SCIROCCO strategy consists of the identification of 30 potentially adoptable GPs in the five regions. Within SCIROCCO good practices are defined as inspiring real-life examples of successfully applied innovations in integrated care. The viability of these GPs is assessed using a form which is developed during the implementation. After which 15 GPS are selected as viable scaling up practices. Second, the maturity requirements for transfer of these 15 GPs are assessed using the SCIROCCO tool (see box 1), resulting in a guide on how to scale up given the local context in which the practices have been developed.

Box 1 Description of the SCIROCCO tool

The SCIROCCO tool consists of 12 dimensions which represent the range of activities that need to be managed in order to deliver integrated care. The maturity of health care system for integrated care or the maturity requirements of GPs in integrated care are assessed by considering each dimension and allocating a measure of progress or 'maturity' (on a 0-5 scale) to each dimension. The scales include the maturity indicators and reflect the basic indications to look for when assessing the current situation of the maturity of health care system for integrated care or the maturity requirements of GPs. After the assessment, a simple graphical representation (i.e. spider diagram) of status can be derived which reveals areas of strength, further attention and improvement in each of the 12 dimensions.

Step 2: Self-assessment process in the five regions

The second step exists of the development of a guide for the regions describing how to use the tool as a self-assessment tool. Hereafter, the five participating European regions assess their maturity in the adoption for integrated care, using the online SCIROCCO tool, to identify strengths, gaps and areas for improvement. Subsequently, the outcomes of the assessments are collected and analysed, thereby informing about the maturity gaps of a regional health and care system in integrated care. The five regions are then clustered in terms of their complementary strengths and weaknesses. Using the outcomes on clustering of regions, the regions are paired in such a way that the knowledge transfer will flow between the regions with the same strengths (twinning) as well as between the regions scoring high at particular dimension with the regions scoring low along the same dimension (coaching).

Step 3: Knowledge transfer processes in the five regions

The last step consists of the development of the process for the twinning and coaching activities of five European regions to support the creation of twinning and coaching relationships by using the information (steps 1 and 2 of SCIROCCO's strategy) to construct these relationships. The process of knowledge sharing and information flow among the five European regions is facilitated and one

For peer review only - http://bmjopen.bmj.com/site/about/guidelines.xhtml

twinning and one coaching activity per region is envisaged. The SCIROCCO tool is used to assist in this process. The twinning and coaching activities are organised as face-to-face meetings, webinars and various other online tools.
The last activity comprises the development of an action plan in each of the five European regions. The action plans reflect the findings of the self-assessment process and specifically focuses on addressing the weaknesses in the maturity of regional health and care system. The action plan informs the decision-makers about the priority actions necessary for improvement of their health and care systems. The actual implementation of the plans and monitoring of their progress (i.e. ongoing self-assessment) is not within the scope of the implementation, due to the limited duration of the SCIROCCO project.

Figure 1 Overview of the three main steps and intended activities per step of the SCIROCCO scaling up strategy

Main scaling up steps of SCIROCCO strategy

**Step**	**1**	**2**	**3**
	Assessment of maturity requirements of GPs* in the five regions	Self-assessment process in the five regions	Twinning and coaching (Knowledge transfer) in the five regions
**Objectives/activities**	a) Identify GPs<br>b) Assess viability GPs and select 15 with the potential for scaling-up<br>c) Assess maturity requirements GPs	a) Develop guide for self-assessment<br>b) Self-assessment in five regions<br>c) Collect and analyse data from the self-assessment process to identify strengths and weaknesses of the five European regions<br>d) Clustering of regions in terms of their complementary strengths and weaknesses	a) Develop the process for twinning and coaching activities<br>b) Facilitate process of knowledge sharing and information flow among five European regions using the tool to facilitate this process<br>c) Develop action plan in each of the five European regions

*GP=Good Practice

Data collection and study population

To assess the fidelity of each scaling-up step, we used a multi-method approach. Data were collected during the implementation of the strategy from each of the partners and local stakeholders involved in implementation of the strategy. Data collection methods included key informant interviews, focus groups, a questionnaire study and the collection of project documents (i.e. progress reports and deliverables). For the reporting of study, we used the consolidated criteria for reporting qualitative research (COREQ) [19].

The extended conceptual framework for implementation fidelity, developed by Hasson [17] and based on the work of Carroll et al. [20], was used for evaluating implementation fidelity of SCIROCCO's step-based approach (the topics are presented in the next section).

The following topics were leading in the data gathering and -analysis the data:

- content (the way in which the steps within the strategy were undertaken and changes in these steps);
- frequency and duration (duration was interpreted by checking the timeline and deadlines for the implementation of the three steps);
- facilitating conditions (quality and usefulness of the protocol, guidance and collaboration within the scaling up strategy);
- context (barriers and facilitators in carrying out the strategy);
- participant recruitment procedures;
- points of improvement.

Qualitative data, including semi-structured interviews and working documents (interim report, final report, project deliverables, local reports and e-mails), were the main data sources to explore

For peer review only - http://bmjopen.bmj.com/site/about/guidelines.xhtml

adherence and possible factors that moderate adherence to the implementation of three steps of the SCIROCCO scaling up strategy. In Table 1 an overview is provided including the examined implementation fidelity components (adherence subcategory and potential moderating factors) in this study, the accompanied research question, data collection procedure/source and planning.

Table 1 Implementation fidelity components (adherence subcategory and potential moderating factors), research question, data collection procedure/source and planning)

**Adherence subcategory**	**Research question(s)**	**Data collection procedures/sources**	**Measurement planning**
Content	How are the three steps of the of scaling up strategy delivered in the five regions?	-Semi-structured interview with supportive and regional partners	Alongside SCIROCCO project<br><br>After the study visits
		-Work documents (i.e. progress reports, interim reports) of supportive and regional partners	Alongside SCIROCCO project
		-Deliverables of supportive and regional partners	Alongside SCIROCCO project
Frequency/Duration	How many GP assessments are performed in the five regions?<br><br>How many self-assessments are performed in the five regions?<br><br>How many twinning and coaching activities are performed in the regions?<br><br>What was the duration of the implementation of the three steps?	-Semi-structured interview with supportive and regional partners<br><br>-Work documents (i.e. progress reports, interim reports) of supportive and regional partners<br><br>-Deliverables of with supportive and regional partners	Alongside the SCIROCCO project
Coverage (Reach)	How many local stakeholders per region participated in the different steps of the SCIROCCO strategy?	-Work documents of regional partners<br><br>-Deliverables of regional partners<br><br>-Emails with regional partners	Alongside program
Potential moderating factors			
Participant responsiveness	How satisfied were the participants with their participation in the study visits? How did the participants	-Focus groups with local stakeholders and regional partners of the five participating regions	After the study visits

For peer review only - http://bmjopen.bmj.com/site/about/guidelines.xhtml

	perceive the outcomes and relevance of the study visits?	-Short survey with the local stakeholders and regional partners of the five regions on experience in in the study visit	
Participant recruitment	What recruitment procedures were used to attract local stakeholders to participate in the three steps of the strategy?	- Semi-structured interview with supportive and regional partners<br><br>-Focus groups with local stakeholders and regional partners of the five participating regions<br><br>-Deliverables with supportive and regional partners	Alongside SCIROCCO program<br><br>After the study visit<br><br>Alongside SCIROCCO program
Conditions to facilitate implementation	What conditions were used to support the implementation of the SCIROCCO scaling up strategy? How were these conditions perceived by SCIROCCO partners and local stakeholders involved in the strategy?	-Semi-structured interview with supportive and regional partners<br><br>-Focus groups with local stakeholders and regional partners of the five participating regions<br><br>-Work documents (i.e. progress reports, interim reports) of with supportive and regional partners<br><br>-Deliverables of with supportive and regional partners	Alongside SCIROCCO project<br><br>After the study visits<br><br>After the last twinning/coaching sessions<br><br>Alongside SCIROCCO project
Context	What factors at political, economical, organizational and work group level affected the implementation of the scaling up strategy?	-Semi-structured interview with supportive and regional partners<br><br>-Focus groups with local stakeholders and regional partners of the five participating regions<br><br>-Work documents (i.e. progress reports, interim reports) of supportive and regional partners<br><br>-Deliverables of supportive and regional partners	After the respective program component is finished<br><br>After the study visits<br><br>Alongside SCIROCCO project<br><br>Alongside SCIROCCO project

The semi-structured interviews were conducted over Skype with members of the SCIROCCO project responsible for the different steps in the strategy (n=12). The interviews were held by the first author and lasted about 60 minutes each. Details on the interviews are presented in Appendix A.

Furthermore, five focus groups were organised after each of the five twinning and coaching study visits (on location) and included the regional SCIROCCO partners in the five regions and the external local members who participated in the study visits (referred to as ‘local stakeholders’). These local stakeholders were recruited by the regional SCIROCCO project partners. The focus groups were alternately facilitated by the first author and two members of work package (WP8) which was part of the SCIROCCO project and focused on collecting lessons learned on the process of using the SCIROCCO tool and strategy. The three female facilitators possessed a minimum of a master’s degree and experience in qualitative research. One of the facilitators holds the position of principal eHealth policy analyst, the other two were researchers working at universities. At the start of the focus groups, the moderators introduced the focus group and themselves, explaining the purpose of the

For peer review only - http://bmjopen.bmj.com/site/about/guidelines.xhtml

focus group, and requesting the participants to sign the informed consent form (see ethics statement). All participants received an overview of the focus group questions at the beginning of each study visit. Data collected from these focus groups focused on the experiences of the participants on the study visits which were organised as part of step three from the scaling up strategy, and included the following topics: content, participant recruitment procedures, participant responsiveness, and points of improvement. The focus groups lasted approximately an hour. In Table 2 an overview of the characteristics of each focus group is provided. Sometimes the same participants were included in the two focus groups. This happened when participants took part of two study visits. The topics of the focus groups were different. The interviews and focus groups were, after obtaining signed consent, audiotaped and transcribed and during both field notes were made.

After the focus groups were conducted, a survey was distributed among the same participants (n=49) to collect data about participants responsiveness (expectations, satisfaction, clarity and usefulness of results of study visit). The characteristics of the participants who completed the survey are shown in Table 3. Some stakeholders participated in two study visits and completed the survey for both visits.

Table 2 Characteristics of the focus groups

**Location**	**Subject of focus group**	**Participants from region (number)**	**Total number of participant s**
1. Puglia	Experience study visit Puglia on GP* in telemonitoring	Experts from Puglia (3), Olomouc (5) and Scotland (6)	14
2.Basque country	Experience study visit Basque country on GP in Advanced Care Planning	Experts from the Basque country (3) and Norrbotten (3)	6
3.Scotland	Experience study visit Scotland on dimension Innovation Management of the SCIROCCO tool	Experts from Scotland (3) and Norrbotten (5)	8
4.Norrbotten	Experience study visit Norrbotten on dimension eHealth & Information services of the SCIROCCO tool	Experts from Norrbotten (4) and Olomouc (4)	8
5.Scotland	Experience study visit on GP in third sector in Scotland	Experts from Scotland (3), Puglia (4) and Basque country (6)	13
		Grand total:	49

*GP= Good Practice

Table 3 Characteristics of participants who completed the survey (n=40)

**Overall**	(n) of participants	
Region of origin participants	Region 1 (Basque Country) = 8<br>Region 2 (Olomouc) = 9<br>Region 3 (Norrbotten) = 10<br>Region 4 (Puglia) = 6<br>Region 5 (Scotland) = 7	
**Per study visit:**		
Puglia (n=12)	Transferring region Puglia=2	Adopting regions: Olomouc=5 and Scotland=5
Scotland (n=6)	Transferring region Scotland=2	Adopting region Norrbotten=4
Basque country (n=6)	Transferring region Basque country=3	Adopting region Norrbotten=3

For peer review only - http://bmjopen.bmj.com/site/about/guidelines.xhtml

Scotland (n=9)	Transferring region Scotland=0	Adopting region Puglia=4 and Basque Country=5
Norrbotten (n=7)	Transferring region Norrbotten=3	Adopting region Olomouc =4

Data analysis

Data from the interviews, focus groups and documents were analysed using content analysis [21]. A coding scheme, including the implementation fidelity concepts and each intervention component, was used during the coding process. The scheme was tested independently by two researchers (LG, HJMV) prior to undertaking the coding process. The analysis of the transcripts and documents was conducted in NVivo 12. The first coder (LG) coded all transcripts using the coding scheme and the second coder (HJMV) operated as a control and coded a random selection of 10% of the transcripts and 10% of the collected documents. The results from this coding process were discussed among the researchers and any disagreement was resolved until consensus was reached. The surveys collected after the twinning and coaching study visits were analysed using descriptive methods in SPSS version 25. Validity of the findings was ensured by using member checks (summaries of the interviews were sent to the respondents, numbers were checked by sending confirmatory emails to the responsible partners) and triangulation (i.e., data from focus groups, documents and interviews were collected to locate/inform the concepts of implementation fidelity) [22], this also ensured data saturation [23].

Results

In this section, first, the results concerning adherence to the three steps of the scaling up strategy during its implementation in the five regions are presented. Here after, the moderating factors which were found to influence the implementation of the scaling up strategy in the five regions are described.

Adherence subcategories

Content

All the steps of the scaling up strategy (maturity requirements in selected GPs, self-assessment process, and twinning and coaching) were implemented with acceptable fidelity. Also, the methodologies developed during the implementation of the steps, which described how to implement the activities in the regions were followed by the regions to a large extent. More details on the content and any deviations observed during the implementation of the steps in the five regions are described below.

Step 1: Maturity assessment of GPs

All the five regions collected data on GPs in their region, implemented the viability assessments of selected GPs and assessed the maturity requirements of the prioritised GPs by applying the tool. A deviation was found in delivering the maturity requirement assessment of the selected GPs in the regions, this assessment was performed twice. This was due to observed heterogeneous outcomes across the five SCIROCCO regions. Also, CHAFEA, the fund holder on behalf of Horizon 2020, requested for performing an extra assessment. In Figure 2, the process of the revised assessment is presented, and all the five regions followed the steps of the revised process in their region. The main difference between the first and second assessment was the number of assessors and focus of the assessment. In the first assessment, the focus was on the maturity of the context wherein the GP was developed and was performed by a single representative of the GP. In the second assessment, a group of experts assessed the GP with the focus on the maturity needed to implement the GP in different health and social care settings.

Figure 2 Revised process on the maturity assessment of Goof Practices (GPs)

For peer review only - http://bmjopen.bmj.com/site/about/guidelines.xhtml

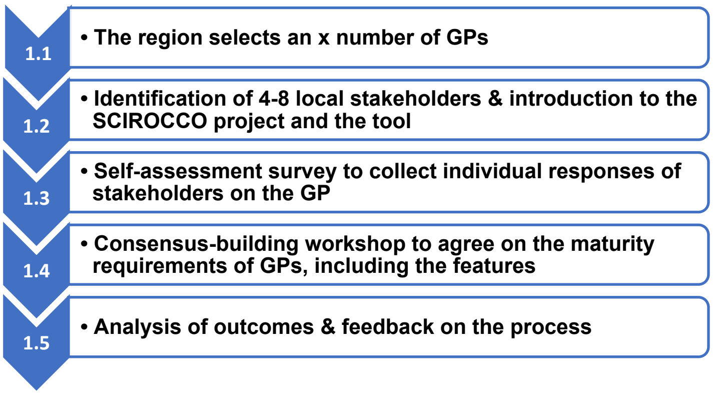

Step 2: Self-assessment in five regions

The five participating regions implemented the self-assessment process according to the developed methodology (as shown in Figure 3) and applied the tool. All regions wrote a report on the implemented local self-assessment process in their region, including an analysis on the strengths and weaknesses and priority actions for their region.

Figure 3 Developed methodology on the maturity assessment of health care systems

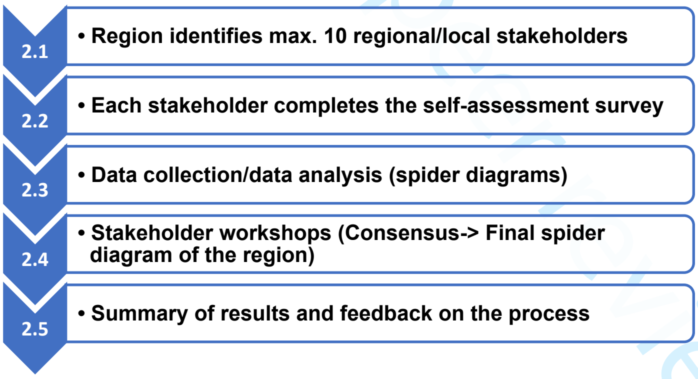

Step 3 Twinning and coaching

Each of the five SCIROCCO regions were involved in the twinning and coaching processes. The process designed to conduct twinning and coaching activities in the regions consisted of three key phases:

Phase 1: Planning for the twinning and coaching;
Phase 2: Knowledge transfer activities;
Phase 3: Capturing the outcomes of twinning and coaching.

The twinning and coaching activities took place between a transferring region and adopting region. The transferring region was the region acting as the “coaching” partner in twinning and coaching activity. The receiving region was the region seeking support and know-how in order to deploy a GP and/or improve a specific aspect of integrated care and acted as the “learning” partner. During implementation of this step, a variation was observed as one region did not play the role of coaching region due to low maturity scoring across all SCIROCCO dimensions. As a result, one region acted twice as transferring region. Another variation was found in the fact that two regions participated twice as receiving region.

In Figure 4, an overview of the process of phase 2 on the knowledge transfer activity is provided. Local variation in the implementation of these steps per twinning and coaching activity were observed. This was because the individual regions were provided the opportunity to reflect their local needs and strategic priorities for integrated care in (the scope of) the twinning and coaching process. The implementation of prior contact between the transferring and adopting regions varied (step 3.3). In addition, some of the documentation which were prepared for the twinning and coaching activity were

For peer review only

For peer review only - http://bmjopen.bmj.com/site/about/guidelines.xhtml

translated. The outline of the program for the study visits also varied per activity (step 3.4) as the different study visits were adapted to the topic of the visit and the needs of the adopting regions. The general outline of the program included presentations of the transferring and/or adopting regions, live demonstrations/site visit of the GP (if relevant) and a facilitated discussion on the maturity requirements using the SCIROCCO tool. During the several study visits, the tool was applied in different ways because the partners were interested to test the use of the tool in the twinning and coaching activity. Furthermore, the tool was not explicitly used in one study visit because of time constraints.

With regard to the last activity, developing the action plans (phase 3), the developed methodology included the following steps: the receiving regions were asked to organise a local meeting in their region reflect on the outcomes of knowledge transfer activities and agree on the local priority actions for the transferability of learning. Here after, the action plans were co-designed by transferring and receiving regions. All five regions participated as receiving regions and wrote an action plan reflecting the outcomes of the twinning and coaching activities. Once completed, the intend was to upload the action plans in the online SCIROCCO tool and share these with all relevant stakeholders. In the last step, the plan was to promote the outcomes of the knowledge transfer activities locally and across the regions, no specific details could be retrieved on the last two intended activities.

Figure 4 Twinning & coaching process

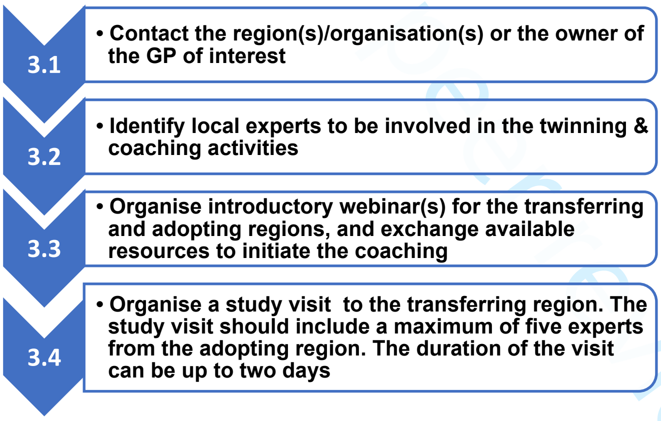

Frequency and duration

In Table 4, a general overview is provided on the frequency of the three SCIROCCO steps, to see whether the target which were set for the different steps were met during the implementation of the strategy. The target number of activities were fully executed during implementation, sometimes even exceeded the target. When looking into the revised maturity assessment of GPs (step 2), one deviation was observed during the second GP assessment of one GP in region 5. During the time of the second assessment the GP was embedded as part of the routine practice at national level, therefore, it was not possible to conduct a second assessment on the GP itself.

Table 4 Frequency of SCIROCCO's activities (figures represent number unless indicated otherwise)

Step	**Activities**		**Project target**	**Delivered**	**Region 1(B)**	**Region 2 (O)**	**Region 3 (N)**	**Region 4 (P)**	**Region 5 (S)**
1	Number of viability assessment of GPs in the five regions		30	32 (106.7%)	7	4	6	8	7
	Number of GPs selected and assessed for maturity assessment	Assessed with first designed methodology	15	15 (115.4%)	3	3	3	3	3
		Assessed with revised methodology (including all steps	15	14 (107.7%)	3	3	3	3	2

For peer review only - http://bmjopen.bmj.com/site/about/guidelines.xhtml

		conducted of the revised process)							
2	Regions performed the complete self-assessment process		5	5 (100%)	1	1	1	1	1
	Completed and documented assessments		5	5 (100%)	1	1	1	1	1
3	Number of regions which performed role as transferring/ coaching region in twinning and coaching activity		≥5	5 (100%)	1	0	1	1	2
	Duration of study visit (up to two days)				1,5 day	0 day	1,5 day	1,5 day	One 1,5 day and one 1 day
	Number of regions which performed the role as adopting region		≥5	7 (140%)	1	2	2	1	1
	Agreed action plans to transfer and/or scale-up interventions		≥5	7 (140%)	1	2	2	1	1

During the implementation of the steps, adjustments were made in the timeline of all the steps. An overview of the original timeline and the executed timeline is provided in Appendix B. A delay was observed in organising the local self-assessment processes in the regions (step 2) since the engagement of the local stakeholders for the self-assessment took much longer than anticipated. In addition, after the self-assessment process, the re-assessment of the maturity requirements of 15 GPs (step 1) needed to be executed in the five regions. Since the planning of the implementation of step 3 was depended on the outcomes of steps 1 and 2, step 3 was organised with a delay of 6 months. The parallel development of SCIROCCO methodology for twinning and coaching allowed the immediate start of the approach in the regions, including the development of regional action plans. In the original program, when the partners were thinking about the description of the steps, what might have been improved was the duration of the strategy which was indicated by one supportive partner.

Coverage

The coverage of the local stakeholders participating in the three main steps of the implemented strategy are shown in Table 5. Since the methodology of the different activities was designed during implementation, the target number for identifying the local stakeholders to participate in the different activities per region was not defined beforehand but were indicated during implementation. These target numbers for the different activities are also presented in Table 5.

For the revised assessment of step 1, the regional SCIORCCO partners were instructed to identify experts with different profiles, backgrounds and experiences from both managerial and practice profile. The regions adapted the identification of experts to their local context/local GPs, which lead to variation in the structure and the number of stakeholders involved per region. For the activities of step 2 and 3, a variation in structure and the size of the local teams of engaged experts per region was also observed. The local variations were the results of the fact that for step 2, the scope of the self-assessment activity was defined by each individual region reflecting the structure of their health care system and the concept of integrated care and the regions were instructed to capture a diversity of perspectives in the assessment process, including different disciplines, sectors and positions in the organisation. For step 3, the scope of the twinning and coaching process was also defined by each individual region reflecting the local need and strategic priorities for integrated care and the regional

For peer review only - http://bmjopen.bmj.com/site/about/guidelines.xhtml

partners selected the stakeholders based on their experiences with the subject of the twinning and coaching activities. This also resulted in variations in the local teams of engaged experts per region.

Table 5 Coverage of local stakeholders per step of the scaling up strategy

Step	Activities	Region 1 (Basque Country)	Region 2 (Olomouc)	Region 3 (Norrbotten)	Region 4 (Puglia)	Region 5 (Scotland)	Total
1	Number of participants assessed the maturity of the context where the GPs* was developed (first methodology)	3x1	3x1	3x1	3x1	3x1	15
	Number of individual questionnaires collected per GP (4-8 experts maximum) (second methodology)	1x4<br>1x4<br>1x4	1x2<br>1x3<br>1x3	1x5<br>1x4<br>1x4	1x5<br>1x5<br>1x6	1x3<br>1x4	56
	Number of participants in face-to-face workshop per GP (second methodology)	1x4<br>1x4<br>1x4	1x2<br>1x3<br>1x3	1x5<br>1x4<br>1x4	1x5<br>1x5<br>1x6	1x3<br>1x4	56
2	Number of stakeholders invited to fill in the questionnaire of the SCIROCCO tool	10	>20	9	11	12	
	Number of individual questionnaires completed (target max. 10 local stakeholders per region)	10	5	7	11	9	42
	Number of participants in face-to-face workshop	9	5	7	11	5	37
3		Study visit Basque Country (GP)	Study visit Norrbotten (dimension)	Study visit Puglia (GP)	Study visit Scotland (GP)	Study visit Scotland (dimension)	
	Number of actively involved participants in the study visit (transferring and receiving region)	15	19	22	14	15	85
	Number of actively involved participants from the adopting region in the study visit (max. 5 per region)	5 (one left early)	4	5 (Olomouc)<br>6 (Scotland)	6 (Basque country)<br>4 (Puglia)	4	34

*GP= Good practie

<u>Moderating factors</u>

Participant recruitment

Recruitment for the GP assessment (Step 1)

The regional SCIROCCO partners were provided instructions on how to perform the assessments of the GPs in their region and how to select and invite the local stakeholders (GP leaders). The recruitment process for the first assessment of the GPs was implemented in all the five regions in line with the instructions provided. The regional SCIROCCO partners invited the practice leaders to participate in the GP assessment, and they received guidelines which described the steps to follow.

In the second assessment, using the revised methodology, the GP assessment process was changed, and the assessment was performed by a multi-disciplinary team rather than single key informant. Again, the regional SCIROCCO partners were introduced to the revised methodology and received new instructions. A slight variation among the regions was observed in the identification procedure of

For peer review only

stakeholders. In four regions the local SCIROCCO project partners identified the local stakeholders themselves (Regions 1, 2, 4 and 5). Another region identified local stakeholders with the support of the local steering group (Region 3), these stakeholders were included in a local project team which participated in various SCIROCCO activities. All the regional SCIROCCO project partners sent an invitation via e-mail to all the identified local stakeholders in the five regions and all experts were invited to use the online version of the SCIROCCO tool to conduct their individual assessments. A slight variation was observed as one of the regions organised a meeting to introduce the project to the stakeholders and explain the assessment process (Region 1).

Recruitment for self-assessment process (Step 2)

All the regional SCIROCCO partners received instructions on how to perform the self-assessment in their regions and how to recruit the local stakeholders including predefined selection criteria. Despite the provided instructions and predefined invitation letter, the regional SCIROCCO partners needed to provide additional efforts to explain to the local stakeholders how to participate in the self-assessment process. As a result, extra informative documents and illustrative videos on how to use the SCIROCCO tool in the self-assessment process were provided. Furthermore, the tool was translated in three additional languages. In the regions different procedures to identify the local experts in the self-assessment process were implemented. In four regions the regional SCIROCCO partners recruited the local experts to participate in the self-assessment activities (Region 1, 2, 4 and 5). Another regional partner identified local stakeholders with the support of the local steering group (Region 3). In addition, the recruitment in the regions varied in using different communication methods and in the length of the process. In some regions, there was a need to clearly communicate the added-value and benefits of the assessment process (Regions 1 and 5 and organised a prior meeting to explain the self-assessment process. One region (Region 4) translated the prepared documents. Another region, called the identified experts by phone (Region 2). In this region, some of the stakeholders expressed that it was hard to respond to the tasks (filling in the on-line tool) as the concept of integrated care is not an urgent topic on the agenda of their country. Subsequently, the recruitment of local stakeholders was a difficult and long procedure for this region. Finally, in some regions it was a challenge for the local experts to reserve time to participate in the activities (Regions 2 and 3).

Recruitment for twinning and coaching (Step 3)

The local experts who participated in the twinning and coaching activities were identified by the regional SCIROCCO partners in all five regions. The recruitment of experts varied per region, in terms of use of different communication channels and in length of the process. Exact details on how the experts were recruited could not be retrieved from the data. A few experts were already involved in previous activities within the SCIROCCO project and most experts were new to the project.

Participant responsiveness

The majority stakeholders were observed to have good rapport with the study visits. Most stakeholders who participated in the study visits indicated that the information provided on the content and process prior to the study visit was clear. Also, most stakeholders agreed that they could ask and discuss what they wanted during the visits and the visits matched with the expectations of most of them. Lastly, the majority of the experts indicated that the content of the study visit should somewhat or moderately influenced decisions in their region (see Table 6 for all details).

Table 6 Experiences of stakeholders about study visit (n=40) (figures in %)

**Question**	**Answer categories**				
	Very unclear	Unclear	Neither clear, nor unclear	Clear	Very clear

For peer review only - http://bmjopen.bmj.com/site/about/guidelines.xhtml

Q1. Prior to the study visit, how clear was the information provided on the content and process of the study visit?	5	0	5	55	35
	Strongly disagree	Disagree	Undecided	Agree	Strongly agree
Q2. Were you able to ask and discuss everything you wanted during the study visit?	0	7.5	10	35	47.5
	Much less than expected	Less than expected	As expected	More than expected	Much more than expected
Q3. How well did the study visit matches your expectations?	0	10	22.5	35	32.5
	Not at all influence	Slightly influence	Somewhat influence	Moderately influence	Extremely influence
Q4. To what extent do you think the content discussed during the study visit should influence decisions in your region? (1 answer missing)	0	7.5	42.5	40.5	7.5

The majority of stakeholders indicated appreciation for the usefulness of the study (explicitly addressed in 4 out of 5 study visits). The sharing of experiences and collaboration during the study visits were explicitly mentioned in two focus groups. A participant mentioned in focus group 4 (FG4) that *''we think you have something you can share with us and something we can learn from you.''* The study visits were considered as an inspiration and regarded as an *'injection of optimism''* (FG1) to improve elements in their own regions. In particular, the onsite experiences were appreciated in three study visits, were practices were visited *''in their real context ''*(FG 2). However, in another study visit a respondent indicated that the visit was a bit less concrete and practical than expected.

The amount of time planned for the study visits was in some cases regarded as sufficient and in other cases a limited amount of time for the study visit was experienced. Some respondents indicated that they did not have enough time to reflect on information which was shared. Another respondent mentioned that prior to the visits, it would be useful to go through the tool and have a meeting or webinar with the transferring regions to have a better understanding of what will be the interest of the visiting regions. When two regions participated as adopting regions in the study visit, a few stakeholders indicated that more time would probably be needed for the study visit. On the site of the study visit organisers (transferring regions) it was indicated in two focus groups that the organisations of the visit might have benefited from more time to prepare, to for example arrange ''even more practical examples'' (FG4) and to be able to *''to have the perfect team in place''*(FG3).

During the study visit, the tool was tested in facilitating the discussion between the regions. The use of the tool during the study visit was regarded in four study visits as a support to facilitate the discussion by providing a structure to the conversation. Not all stakeholders had experience using the SCIROCCO tool in the study visits. Two respondents explicitly mentioned that they thought the tool was difficult to understand the first time. A difficulty experienced with the tool was mentioned in the language of the tool, as it was not regarded as the simplest English. According to a stakeholder the terms used in the tool need to be locally interpreted, *''as it should be translated into a local terminology and context''* (FG3). Furthermore, it was indicated by a few stakeholders that having a translator present during the study visit was critical.

Conditions to facilitate implementation

Project coordination, effective communication and guidelines

The central (strategy) coordination was regarded to be a supportive factor to implement the steps of the strategy in the five regions (according to all partners). At the start of the strategy, bi-weekly telecom conferences (virtual meetings) took place over the course of the entire implementation period to facilitate effective communication with the partners. During these telecoms, among other things, the draft methodologies designed for the scaling up steps were discussed. Several additional online

For peer review only - http://bmjopen.bmj.com/site/about/guidelines.xhtml

meetings within the consortium were organised to make sure that all partners understood how to implement the different steps in their region. In addition, five project assembly meetings were organised were the partners were given the opportunity to share and discuss their work on implementing the activities in the five regions. Furthermore, written guidelines on how to implement the different activities in the regions were also shared by email to the regional partners. In addition, three sets of detailed guides were designed to guide the use of the online SCIROCCCO tool. The full set of instructions was also included directly in the online version of SCIROCCCO tool. In addition, several supportive training materials and demo videos were developed an could be accessed online. The available methodology was regarded to be helpful in providing consistency in the self-assessment processes and the twinning and coaching process.

Flexible approach

The *flexible approach* within the strategy to develop the exact design of the steps of the strategy during the implementation and the opportunity to sometimes deviate from the original plan was regarded as a facilitator for implementation as indicated by the regional partners of two regions. One regional partner in interview 7 (I7) indicated: *''you give kind of an idea on how it should work, but then in real life things happen in a different way.''* One regional partner indicated that the flexible/open approach for developing the steps could have influenced the planning of the strategy, because in-depth discussions among the consortium partners or the representatives of regions sometimes took more time than anticipated.

Supportive attitude of regional partners

The exact design of the steps of the strategy were developed in collaboration with the support of other work packages and the project coordination. The collaboration among the different regional and supportive partners was regarded as open and a positive experience which was explicitly mentioned by four partners. A facilitator for implementation mentioned by two regional partners was regarded in the commitment of all the project partners to achieve the objectives of the project. Also, one partner further indicated that the flexible attitude of all project partners was regarded to facilitate implementation.

Regional conditions

The regional partners of two regions indicated that they had several co-workers from their region involved in the project which supported the implementation of the project activities in their region. For example, Region 3 had set-up a local steering group, which appointed people to be part of a local project team, including people with different roles and responsibilities. This local working group worked on the activities of the SCIROCCO project and from this group some stakeholders were also recruited to participate as the stakeholder team throughout the project.
Lastly, before step 2 was implemented in all the five regions, the designed process was pilot tested in one region. One regional partner expressed that this was regarded as facilitative for the implementation in the other regions. For the implementation of the GP assessment (step 1) the regional partner of another region indicated that it perhaps would have been good to also pilot test the method in their own region before other regions followed.

Context

The different regional contexts of the five SCIROCCO regions were found to influence the implementation of the steps in the five regions. Several context factors may have moderated adherence to the various scaling up steps.

Language and conceptual adaptations on integrated care

During implementation, an unanticipated activity was the translation of the tool to improve stakeholders' experience with the tool. In addition, the provided invitation letters needed to be translated and all the individual assessments (for step 1 and 2) as well as consensus-building workshops were held in local languages except for one region which was also held the workshop in English (Region 3). During the implementation of the project it was observed that the translation of the

For peer review only - http://bmjopen.bmj.com/site/about/guidelines.xhtml

tool is not enough. To improve the understanding of the tool, it was indicated that the concepts of the tool needed to be adjusted to local linguistic and contextual aspects of integrated care. However, this cross-cultural adaptation was found not to be feasible in the duration of the strategy and the recommendation was that this should be considered as a potential improvement of the tool in the future.

Level of development in integrated care

The variation of the regions in the level of development and implementation of IC influenced the implementation. During the implementation of the SCIROCCO steps 2 and 3, the five regions were able to define the scope for the self-assessment process and the twinning and coaching activities to reflect the structure of their healthcare systems, the concept of integrated care (step 2) and the local needs and strategic priorities for integrated care (step 3). In one region, Region 2, the concept of integrated of care was relatively new. For this region, IC is not widely known, and stakeholders have limited knowledge about nor experience with this approach. Hence, the assessment processes proved to be a complex task for the region, especially in the engagement with local stakeholders. Also, the region did not play the role of transferring region due to low maturity scoring across all SCIROCCO dimensions. Furthermore, it was indicated by one partner that in the regions where the IC agenda was strongly established, the implementation of the activities of the project ran smoother.

Local perspectives

The variation in implementing the steps may have also been influenced by the different perspectives of the SCIROCCO partners in the regions. One supportive partner mentioned that the regions varied in their perspectives as some were more policy driven, some more practical or research focused, which could have influenced the implementation, since the implementation of step 2 and 3 were adapted to the regional context and needs.

Changes in local environment

During implementation of SCIROCCO, changes in the local environment of the different participating regions were indicated to possibly influence implementation. Some organisational changes occurred in the public authorities were the regional partners were affiliated. For one region the focus of their health system agenda changed. In another region, the change in management of their organisation influenced how well known the project was and how much leverage the project had. Another regional change during implementation was observed on the level of a GP. One GP was embedded as part of the routine practice at national level. As such, this GP could not be included in the second assessment on the GP in step 1.

Discussion

This study assessed the implementation fidelity of a step-based scaling up strategy for IC which was implemented in five European regions as part of a cross national project. The results show that all steps of the scaling up strategy were implemented with acceptable fidelity. All five regions conducted the GP self-assessments (step 1) and undertook the self-assessment process of their health care system context in their regions (step 2). Each region also participated in the twinning and coaching process and wrote one or two action plans (step 3). In addition, the targets which were set for each step were met or even exceeded (frequency). However, some deviation was also found. First, the GP assessments in the regions were implemented twice (step 1) and one region did not participate as transferring region in the twinning and coaching activity (step 3). Also, the duration of the steps 1 and 2 took longer than anticipated. Lastly, the coverage of the local experts per region who participated in the three scaling up steps varied in structure and size.

We found several factors that influenced implementation fidelity of the scaling up strategy. These included facilitating conditions in the flexible approach and coordination, positive participant responsiveness, regional variability in participant recruitment procedures and several contextual factors. Supportive influencing factors for implementation of the three steps in the five regions were found in good coordination to implement the steps, the use of effective communication strategies among the partners, the provision of guidelines on how to implement the processes and the

For peer review only - http://bmjopen.bmj.com/site/about/guidelines.xhtml

SCIROCCO tool in the regions. Furthermore, the flexible approach, the supportive attitude of partners, and certain regional conditions (i.e. having a local steering group and regional pilot testing) have facilitated the implementation of the steps in the regions. A last important factor was the positive experience of the local stakeholders which participated in the study visits.

Several factors found in facilitating conditions (the flexible approach), the local variations and adaptions in participant recruitment procedures and several contextual factors moderated adherence to the implementation of the strategy. The contextual factors which influenced the implementation of the strategy were reflected in the local differences in language and conceptual understanding of integrated care among the regions. In addition, the different health care system contexts, level of development in IC, perspectives of the SCIROCCO partners in the regions and the local needs and strategic priorities resulted in variations in implementation of the strategy. Lastly, regional changes during the implementation of the strategy also influenced adherence. For example, for step 2 a selected GP could not be included in the second assessment as, during the lifespan of the strategy, it was embedded as part of the routine practice at the national level.

The implementation of the strategy consisted of a partly standardised and partly flexible approach. Flexible interventions present a challenge for evaluators, as flexibility and fidelity can be at odds with each other. The flexible approach to adapt or deviate from protocols is in contrast with examining the implementation fidelity of an intervention to its protocol. The discussions about the conflict on how to consider fidelity and the extent to which adaptation across contexts is fair or needed are unresolved [24]. However, adapting health service delivery to local conditions has several advantages. The variety in implementation found in our study corresponds to the recognition by Carroll et al., that an ''intervention cannot always be implemented fully in the real world. Local conditions may require it to be flexible and adaptable [16].'' Furthermore, it has the potential to improve program effectiveness by means of increased program feasibility and increased user adoption [20]. In such a case, it is important to take into account flexibility during implementation because of needed adaption to the local conditions. This occurred within SCIROCCO as the scope of the activities were informed by each individual region reflecting the structure of their healthcare systems, the framework of IC and the local need and strategic priorities for integrated care. To draw valid conclusions about the fidelity of an implementation, it is important to accomplish a good balance between adherence to a protocol on the one hand and the required flexibility for adaptations on the other hand.

To our knowledge, this study is the first to assess the fidelity of the implementation of an international scaling up strategy for integrated care. Most studies which assess the implementation fidelity focus on the implementation of national (complex health) interventions or programs [25–27]. Consequently, we were not able to compare our findings to similar studies. Notwithstanding, our study shows that assessing the implementation of an international step-based scaling up strategy by using the modified version of the Conceptual Framework for Implementation Fidelity [17] is feasible. Hence, we encourage others who work or plan to work on similar strategies to study the implementation fidelity of such strategies, to clarify the elements that are associated with the implementation of their strategy.

Nolte et al. explored the national experiences with developing, implementing and impacting the wider system context of integrated care in three countries [28]. They concluded that ''a more formal strategy'' for expansion is needed, as the wider dissemination of the projects studied occurred in ''an incremental and somewhat haphazard way''[28]. The scaling up strategy presented in this study focuses on the adoption of integrated care initiatives to other country settings, which is challenging because of different contexts. The scaling up strategy provides an encouraging example of a structured approach to scaling up of integrated care initiatives in an international context. The results of this study provide insight in the factors which need to be considered when implementing the strategy.

Strengths and limitations

The opportunity to assess the implementation of the SCIROCCO strategy in its real-life setting of five European regions enabled us to collect several types of data, to triangulate data, and hence enhance in-depth insight into the implementation. Furthermore, our role within the project provided a unique opportunity to obtain a close collaboration with the partners to collect the data needed for the implementation fidelity assessment. However, being a partner in the consortium responsible for

For peer review only - http://bmjopen.bmj.com/site/about/guidelines.xhtml

undertaking the evaluation activities, the subjective experiences of the participants (supportive partners, regional partners and local stakeholders) could have been influenced by social-desirability and/or recall bias.

We were aware of our role and to safeguard objectivity we did not interfere with implementation of the project. However, due to time constraints and a high demand on the participants, we were not able to collect data on all pre-planned moments which limited the study coverage of the potential factors influencing the implementation of complex interventions. Only data from one regional partner could be collected during implementation, whereas the rest of the data were collected at the end of the implementation. This limited our insight into the experiences and involvement of participants in other steps. To be able to explain ratings more in-depth we recommend future researchers to examine participant experiences at several moments during the process of implementation. In addition, we have no specific information about the non-responders of the focus groups, since local stakeholders were recruited by the regional SCIROCCO partners we did not have insight in how many local stakeholders were approached by the partners. Therefore, we were unable to track the response rate in the study. Several factors may have contributed to non-responses, which could be the time demands on some respondents of participating in the SCIROCCO project itself (it took at least two full days and traveling time) or not seeing the immediate benefit from participating.

Due to the nature of the strategy, where the methodologies of the steps were developed during the implementation (including identification of the number of stakeholders), measurement of adherence was challenging. This corresponds to other studies who also explored the implementation fidelity with a lack of defined points of references for assessment [25,26,29]. The opportunity to work closely with program implementers ensured us to be kept up to date on the changes regarding revisiting reference points with the evolution of the strategy.

Conclusion

Overall, the SCIROCCO strategy was regarded an encouraging approach offering regions a tailored but flexible path intended to facilitate progress in integrated care. The multi-method design of this study has yielded knowledge about what elements are involved in implementing a European scaling up strategy concerning IC initiatives. The use of a theoretical framework helped us to document the process by which the strategy was implemented and understand the level of implementation fidelity achieved. The insights obtained could support other regions, not being part of the SCIROCCO project, on what to consider when interested to use the tool and processes to achieve knowledge transfer with other regions to ultimately scale-up IC initiatives. Implementing the strategy might be challenging for regions which are less known to concept of integrated care and when the strategy gets modified depending on the local context of regions. Furthermore, the implementation of the steps in the five regions and this evaluation study took place within the boundaries of a project, where dedicated resources were provided. An important question is whether the SCIROCCO strategy will be as successful without these available resources and whether the evaluation study will encounter other factors that apply to the implementation in these settings. To test the external validity of study findings, it would be recommended to also test the SCIROCCO strategy in other countries both in- and outside Europe. Furthermore, lessons on implementation of the developed action plans would also be useful for other interested regions.

Footnotes

Abbreviations

IC: integrated care, GP: good practice, SCIROCCO: Scaling Integrated Care in Context.

Ethics approval and consent to participate

The conduct of this study was granted permission by the Institutional Review Board of the Vrije Universiteit Brussel (reference: B.U.N. 143201734384). Participant consent was requested before the interviews and focus groups in the form of an informed consent. Signed written consent forms were completed by all participants.

Consent for publication

Not applicable.

Availability of data and material

For peer review only - http://bmjopen.bmj.com/site/about/guidelines.xhtml

The datasets used and analysed during the current study are available from the corresponding author on reasonable request.

Competing interests
The authors declare that they have no competing interests.

Funding
This study was undertaken within the Scirocco project which was co-funded by the Health Programme of the European Union under Grant Agreement 710033 (CHAFEA). The funding body was neither involved in the design of the study nor the collection, analysis, and interpretation of data and writing of the manuscript.

Authors' contributions
LG and HJMV provided valuable contributions to the design of the work and provided substantial contributions to the analysis of the work. Both authors provided significant contributions to the acquisition of data, interpretation of data and writing of the manuscript. DV and INF reviewed the manuscript critically. All authors read and approved the final manuscript.

Acknowledgements
We would like to thank all SCIROCCO project partners and local experts for their time and valuable contribution in the study.

Reference

1 Gröne O, Garcia-Barbero M. Integrated care: a position paper of the WHO European Office for Integrated Health Care Services. *Int J Integr Care* 2001;**1**:e21.

2 Amelung V, Stein V, Goodwin N, *et al. Handbook Integrated Care*. Springer International Publishing 2017. doi:10.1007/978-3-319-56103-5

3 Nolte E, Pitchforth E. What is the evidence on the economic impacts of integrated care? 2014.

4 Bautista MAC, Nurjono M, Lim YW, *et al.* Instruments Measuring Integrated Care: A Systematic Review of Measurement Properties. *Milbank Q* 2016;**94**:862–917. doi:10.1111/1468-0009.12233

5 Busetto L. *Great Expectations: The Implementation of Integrated Care and Its Contribution to Improved Outcomes for People with Chronic Conditions*. Int. J. Integr. Care. 2017;**16**. doi:10.5334/ijic.2555

6 Goodwin N. Improving Integrated Care: Can Implementation Science Unlock the 'Black Box' of Complexities? *Int J Integr Care* 2019;**19**. doi:10.5334/ijic.4724

7 Hunter A, Brewer J. Designing Multimethod Research. In: Hesse-Bibe S, Johnson R, eds. *The Oxford Handbook of Multimethod and Mixed Methods Research Inquiry*. London: : Oxford University Press 2016. 85–205.

8 Chambers DA, Glasgow RE, Stange KC. The dynamic sustainability framework: Addressing the paradox of sustainment amid ongoing change. *Implement Sci* 2013;**8**:117. doi:10.1186/1748-5908-8-117

9 Lamont T, Barber N, Pury J de, *et al.* New approaches to evaluating complex health and care systems. *BMJ* 2016;**154**:i154. doi:10.1136/bmj.i154

10 Drabble SJ, O'Cathain A. Moving from Randomized Controlled Trials to Mixed Methods Intervention Evaluation. In: *The Oxford Handbook of Multimethod and Mixed Methods Research Inquiry*. London: : Oxford University Press 2015. 406–2.

11 Berwick DM. The Science of Improvement. *JAMA* 2008;**299**:1182. doi:10.1001/jama.299.10.1182

12 Henderson D, Pavlickova A, Lewis L. Scalability and transferability of good practices in Europe: What does it take? *Int J Integr Care* 2016;**16**:1–2.

For peer review only - http://bmjopen.bmj.com/site/about/guidelines.xhtml

13 Dusenbury L. A review of research on fidelity of implementation: implications for drug abuse prevention in school settings. *Health Educ Res* 2003;**18**:237–56. doi:10.1093/her/18.2.237

14 Mccormack B, Kitson A, Rycroft-malone J. Getting evidence into practice : the meaning of ' context '. *Jan* 2001;:94–104.

15 Greenhalgh T, Robert G, Bate P, *et al.* How to spread good ideas: A systematic review of the literature on diffusion, dissemination and sustainability in health service delivery and organisation. *Milbank Q* 2004;:426. doi:10.1111/j.0887-378X.2004.00325.x

16 Carroll C, Patterson M, Wood S, *et al.* A conceptual framework for implementation fidelity. *Implement Sci* 2007;**2**:40. doi:10.1186/1748-5908-2-40

17 Hasson H. Systematic evaluation of implementation fidelity of complex interventions in health and social care. *Implement Sci* 2010;**5**:67. doi:10.1186/1748-5908-5-67

18 Grooten L, Alexandru C-A, Alhambra-Borrás T, *et al.* A scaling-up strategy supporting the expansion of integrated care: a study protocol Article information. Published Online First: 2018. doi:10.1108/JICA-04-2018-0029

19 Tong A, Sainsbury P, Craig J. Consolidated criteria for reporting qualitative research (COREQ): A 32-item checklist for interviews and focus groups. *Int J Qual Heal Care* 2007;**19**:349–57. doi:10.1093/intqhc/mzm042

20 Carroll C, Patterson M, Wood S, *et al.* A conceptual framework for implementation fidelity. *Implement Sci* 2007;**2**:40. doi:10.1186/1748-5908-2-40

21 Weber RP. *Basic content analysis.* Beverly Hills, CA: : Sage 1990.

22 Creswell JW, Miller DL. in Qualitative Inquiry. *Theory Pract* 2000;**39**:124–30.

23 Fusch PI, Ness LR. Are We There Yet? Data Saturation in Qualitative Research. *Qual Rep* 2015;**20**:1408–16.https://nsuworks.nova.edu/cgi/viewcontent.cgi?article=2281&context=tqr (accessed 14 Oct 2019).

24 Moore G, Audrey S, Barker M, *et al.* Process evaluation of complex interventions. *DecipherUkNet* 2014;:19–75. doi:http://www.populationhealthsciences.org/MRC-PHSRN-Process-evaluation-guidance-final-2-.pdf

25 Nurjono M, Shrestha P, Ang IYH, *et al.* Implementation fidelity of a strategy to integrate service delivery: learnings from a transitional care program for individuals with complex needs in Singapore. *BMC Health Serv Res* 2019;**19**. doi:10.1186/s12913-019-3980-x

26 Muntinga ME, Van Leeuwen KM, Schellevis FG, *et al.* From concept to content: assessing the implementation fidelity of a chronic care model for frail, older people who live at home. *BMC Health Serv Res* 2015;**15**:18. doi:10.1186/s12913-014-0662-6

27 Stijnen M, Jansen M, WJ, *et al.* Nurse-led home visitation programme to improve health-related quality of life and reduce disability among potentially frail community-dwelling older people in. *BMC Fam Pract* 2014;:1–14.

28 Nolte E, Frølich A, Hildebrandt H, *et al.* Implementing integrated care: A synthesis of experiences in three European countries. *Int J Care Coord* 2016;**19**:5–19. doi:10.1177/2053434516655626

29 Hasson H, Blomberg S, Duner A. Fidelity and moderating factors in complex interventions: a case study of a continuum of care program for frail elderly people in health and social care. *Implement Sci* 2012;**7**:1–11. doi:Artn 23\rDoi 10.1186/1748-5908-7-23

For peer review only - http://bmjopen.bmj.com/site/about/guidelines.xhtml

For peer review only

For peer review only - http://bmjopen.bmj.com/site/about/guidelines.xhtml

Appendix A Details of the interviews

Participants	Main theme discussed	Time of interview
Project members responsible for the coordination of the project (2 members)	Views and experiences in undertaking the work regarding the coordination of the project.	August 2018
Project members responsible for implementing the first step of the strategy (3 members)	Views and experiences in undertaking the work regarding identification of maturity requirements of selected local IC interventions (GPs) which have the potential for scaling up	First interview in June 2017; second interview on revised methodology September 2018
Project members responsible for the developments of the tool within the strategy (2 members)	Views and experiences in undertaking the work on the refinement of MM	August 2018
Project members responsible for the implementation of the second step in the strategy (2 members)	Views and experiences in undertaking the work regarding the self-assessment process of SCIROCCO regions	July 2018
Project members responsible for the implementation of the third step in the strategy (2 members)	Views and experiences in undertaking the work regarding coaching and twinning of regions	July 2018
Project members responsible for the collecting lessons learned on using the tool and the process of scaling up (1 member, and 1 member provided feedback to the summary of interview)	Views and experiences in undertaking the work regarding the collection lessons learned on the process of scaling up	July 2018

For peer review only

For peer review only

Appendix B Timetable planning project and executed activities

Blue: anticipated duration of task. Orange: actual duration of task spent during implementation as indicated in reports. Yellow: deviated actual duration spent as indicated by other reports.

	1									2												3											4
Month	Apr 16 (M1)	May 16 (M2)	June 16 (M3)	July 16 (M4)	August 16(M5)	Sep 16 (M6)	Oct 16 (M7)	Nov 16 (M8)	Dec 16 (M9)	Jan 17 (M10)	Feb 17 (M11)	Mar 17 (M12)	Apr 17 (M13)	May 17 (M14)	June 17 (M15)	July 17 (M16)	Aug 17 (M17)	Sep 17 (M18)	Oct 17 (M19)	Nov 17 (M20)	Dec 17 (M21)	Jan 18 (M22)	Feb 18(M23)	Mar 18 (M24)	Apr 18 (M25)	May 18 (M26)	June 18 (M27)	July 18 (M28)	Aug 18 (M29)	Sep 18 (M30)	Oct 18 (M31)	Nov 18 (M32)	
Step 1 Maturity requirements in GPs in five regions																																	
Viability assessment of GP																																	
Data collection																																	
Maturity requirements																																	
							1st																2nd										
Step 2 Self-assessment process in five regions																																	
Self-assessment process in five European regions																																	
Strengths and weaknesses of the European regions in integrated care																																	

Step 3 Twinning and coaching activities in five regions																																	
Development of methodology for twinning and coaching																																	
Coaching and twinning acitvities																																	
Development of Action Plans																																	

For peer review only

COREQ (COnsolidated criteria for REporting Qualitative research) Checklist

.

Topic	Item No.	Guide Questions/Description	Reported on Page No.
**Domain 1: Research team and reflexivity**			
Personal characteristics			
Interviewer/facilitator	1	Which author/s conducted the interview or focus group?	8
Credentials	2	What were the researcher's credentials? E.g. PhD, MD	8
Occupation	3	What was their occupation at the time of the study?	8
Gender	4	Was the researcher male or female?	8
Experience and training	5	What experience or training did the researcher have?	8
Relationship with participants			
Relationship established	6	Was a relationship established prior to study commencement?	8
Participant knowledge of the interviewer	7	What did the participants know about the researcher? e.g. personal goals, reasons for doing the research	8
Interviewer characteristics	8	What characteristics were reported about the inter viewer/facilitator? e.g. Bias, assumptions, reasons and interests in the research topic	19
**Domain 2: Study design**			
Theoretical framework			
Methodological orientation and Theory	9	What methodological orientation was stated to underpin the study? e.g. grounded theory, discourse analysis, ethnography, phenomenology, content analysis	9
Participant selection			
Sampling	10	How were participants selected? e.g. purposive, convenience, consecutive, snowball	8
Method of approach	11	How were participants approached? e.g. face-to-face, telephone, mail, email	15
Sample size	12	How many participants were in the study?	8 and 9
Non-participation	13	How many people refused to participate or dropped out? Reasons?	19
Setting			
Setting of data collection	14	Where was the data collected? e.g. home, clinic, workplace	8
Presence of non-participants	15	Was anyone else present besides the participants and researchers?	8
Description of sample	16	What are the important characteristics of the sample? e.g. demographic data, date	8 and 9
Data collection			
Interview guide	17	Were questions, prompts, guides provided by the authors? Was it pilot tested?	8
Repeat interviews	18	Were repeat inter views carried out? If yes, how many?	NA
Audio/visual recording	19	Did the research use audio or visual recording to collect the data?	8
Field notes	20	Were field notes made during and/or after the inter view or focus group?	8
Duration	21	What was the duration of the inter views or focus group?	8
Data saturation	22	Was data saturation discussed?	9
Transcripts returned	23	Were transcripts returned to participants for comment and/or	9

For peer review only

Topic	Item No.	Guide Questions/Description	Reported on Page No.
		correction?	
**Domain 3: analysis and findings**			
Data analysis			
Number of data coders	24	How many data coders coded the data?	9
Description of the coding tree	25	Did authors provide a description of the coding tree?	9
Derivation of themes	26	Were themes identified in advance or derived from the data?	9
Software	27	What software, if applicable, was used to manage the data?	9
Participant checking	28	Did participants provide feedback on the findings?	NA
Reporting			
Quotations presented	29	Were participant quotations presented to illustrate the themes/findings? Was each quotation identified? e.g. participant number	15 and 16
Data and findings consistent	30	Was there consistency between the data presented and the findings?	9
Clarity of major themes	31	Were major themes clearly presented in the findings?	9-18
Clarity of minor themes	32	Is there a description of diverse cases or discussion of minor themes?	18

Developed from: Tong A, Sainsbury P, Craig J. Consolidated criteria for reporting qualitative research (COREQ): a 32-item checklist for interviews and focus groups. *International Journal for Quality in Health Care*. 2007. Volume 19, Number 6: pp. 349 – 357

For peer review only

BMJ Open

Assessment of the implementation fidelity of a strategy to scale-up integrated care in five European regions: a multi-method study

Journal:	*BMJ Open*
Manuscript ID	bmjopen-2019-035002.R1
Article Type:	Original research
Date Submitted by the Author:	04-Feb-2020

Complete List of Authors:	Grooten, Liset; Vrije Universiteit Brussel, Family Medicine and Chronic Care<br>Fabbricotti, Isabelle; Erasmus University Rotterdam, Erasmus School of Health Policy & Management<br>Devroey, Dirk; Vrije Universiteit Brussel, Family Medicine and Chronic Care<br>Vrijhoef, Hubertus; Maastricht Universitair Medisch Centrum+, Patient & Care; Panaxea

**Primary Subject Heading**:	Health services research
Secondary Subject Heading:	Health policy, Research methods
Keywords:	Organisation of health services < HEALTH SERVICES ADMINISTRATION & MANAGEMENT, Organisational development < HEALTH SERVICES ADMINISTRATION & MANAGEMENT, Change management < HEALTH SERVICES ADMINISTRATION & MANAGEMENT

SCHOLARONE™
Manuscripts

I, the Submitting Author has the right to grant and does grant on behalf of all authors of the Work (as defined in the below author licence), an exclusive licence and/or a non-exclusive licence for contributions from authors who are: i) UK Crown employees; ii) where BMJ has agreed a CC-BY licence shall apply, and/or iii) in accordance with the terms applicable for US Federal Government officers or employees acting as part of their official duties; on a worldwide, perpetual, irrevocable, royalty-free basis to BMJ Publishing Group Ltd ("BMJ") its licensees and where the relevant Journal is co-owned by BMJ to the co-owners of the Journal, to publish the Work in this journal and any other BMJ products and to exploit all rights, as set out in our licence.

The Submitting Author accepts and understands that any supply made under these terms is made by BMJ to the Submitting Author unless you are acting as an employee on behalf of your employer or a postgraduate student of an affiliated institution which is paying any applicable article publishing charge ("APC") for Open Access articles. Where the Submitting Author wishes to make the Work available on an Open Access basis (and intends to pay the relevant APC), the terms of reuse of such Open Access shall be governed by a Creative Commons licence – details of these licences and which Creative Commons licence will apply to this Work are set out in our licence referred to above.

Other than as permitted in any relevant BMJ Author's Self Archiving Policies, I confirm this Work has not been accepted for publication elsewhere, is not being considered for publication elsewhere and does not duplicate material already published. I confirm all authors consent to publication of this Work and authorise the granting of this licence.

For peer review only

Assessment of the implementation fidelity of a strategy to scale-up integrated care in five European regions: a multi-method study

Liset Grooten, MSc, Department of Family Medicine and Chronic Care, Vrije Universiteit Brussel, Brussels, Belgium (fennechien.grooten@vub.be, corresponding author)

Isabelle Natalina Fabbricotti, PhD, Erasmus School of Health Policy & Management, Erasmus University Rotterdam, (fabbricotti@eshpm.eur.nl)

Dirk Devroey, PhD, Department of Family Medicine and Chronic Care, Vrije Universiteit Brussel, Brussels, Belgium (Dirk.Devroey@vub.be)

Hubertus Johannes Maria Vrijhoef, PhD, Department Patient & Care, Maastricht University Medical Center, Maastricht, the Netherlands; Panaxea B.V., Amsterdam, the Netherlands; (bert.vrijhoef@panaxea.eu)

Corresponding author

Liset Grooten, MSC

Department of Family Medicine and Chronic Care

Faculty of Medicine and Pharmacy

Vrije Universiteit Brussel

P.O. 103, B-1090 Brussels, Belgium

Tel.: + 32 2 477.43.11

Fax.: + 32 2 477.43.01

Fennechien.grooten@vub.be

Abstract

For peer review only

For peer review only - http://bmjopen.bmj.com/site/about/guidelines.xhtml

Objective The SCaling IntegRated Care in COntext (SCIROCCO) project tested a step-based scaling up strategy to explore what and how to scale-up integrated care initiatives in five European regions. To gain a profound understanding of which factors influence the implementation of this strategy, the objective of this study was to assess the extent to which the SCIROCCO strategy was implemented as planned.

Design Multi-method study

Methods The extended version of the conceptual framework for implementation fidelity was used to evaluate what factors influence the implementation of the scaling up strategy. Data were collected in the five participating European regions during the intervention period. Data collection methods included: key informant interviews, focus groups, questionnaire studies, and project documents.

Results All three main steps of the scaling up strategy were implemented with acceptable fidelity. Variations were observed in the duration of implementing the steps in the regions. Also, variations were observed in the coverage of experts to participate in the steps of the strategy. Several factors were observed to influence the implementation: facilitation conditions (i.e. good coordination for implementation) and participant responsiveness (i.e. a positive experience of participants in the organised study visits). Factors which may have moderated adherence to the original plan of the strategy were found in facilitating conditions (i.e. in the flexible approach), participant recruitment factors (i.e. adaptions of the procedure by the regions) and contextual factors (i.e. the level of development of integrated care).

Conclusion This was the first study to assess implementation fidelity of a European project which used a step-based scaling up strategy in five European regions. Similar European projects that are based on collaboration between several European regions can learn from the lessons captured in SCIROCCO and can become more aware of the facilitating factors and pitfalls of implementing such projects.

Key words: Implementation fidelity, integrated care, scaling up

Strengths and limitations of this study:

- This study was the first to assess the extent to which a step-based scaling up strategy in five European regions was implemented as planned within a European project.
- All five components of the scaling up strategy were implemented with acceptable fidelity. The insights obtained could support other regions interested to use the SCIROCCO tool and processes for achieving knowledge transfer and ultimately scale-up their integrated care initiatives.
- Direct involvement as researchers in this EU project provided a unique opportunity to follow the project closely, and to obtain solid cooperation to conduct the research activities. However, choices needed to be made regarding the data collection to align the research activities within the larger EU project and to be feasible to undertake it within the project time.

Background

For peer review only - http://bmjopen.bmj.com/site/about/guidelines.xhtml

Numerous integrated care initiatives have been developed around the world to bring about accessible, high quality, effective and sustainable health and social care [1]. Consequently, valuable lessons on the design and implementation of the transformation towards integrated care have been gained [2]. However, less known is what factors contribute to the progress and success of integrated care (IC) initiatives. Obtaining such insights is challenging, which is partially induced by deficient and absent evaluation and measurement of IC initiatives, resulting in the lack of evidence on the working mechanisms in IC [3]. In addition, many of the available assessment tools for IC have been poorly or insufficiently validated [4]. Furthermore, in the presence of innumerable models of IC, there is no universal conceptual understanding of integrated care, which leads to a lack of understanding on what 'integration' might variously look like. As a result, insight in how, when and why integrated care initiatives achieve certain results is blurry [5,6]. This lack of knowledge means that it is hard to learn from experiences [7–9] and to know to what extent and in which form integrated care initiatives can be implemented in different settings [10,11].

To explore how to learn from experiences and knowledge of current integrated care initiatives, the Scaling IntegRated Care in Context (SCIROCCO) project tested a structured approach intended to facilitate upscaling of successful initiatives. This EU subsidised project did so by focusing on the context and environment (i.e. the regional delivery system and political and organisational environment) of IC initiatives. A step-based scaling up strategy was implemented as part of the project with the aim to facilitate the implementation of Good Practices (GPs) in IC at local, regional or country level by recognising the maturity requirements of GPs and health and care systems in order to achieve scaling up and knowledge transfer amongst European Member States [12]. The strategy was implemented in five regions (Basque Country (Spain), Norrbotten (Sweden), Puglia (Italy), Olomouc (Czech Republic) and Scotland (United Kingdom).

The SCIROCCO step-based strategy consists of multiple components which are planned to be implemented in five different settings. The implementation of complex and multi-component interventions rarely happens as planned [13]. A variety of factors can influence the implementation of a strategy (like SCIROCCO's), such as the context in which an intervention is implemented [14] and the complexity of the intervention [15]. The local contexts of the different regions in SCIROCCO might have led to necessary adaptations during the implementation of the scaling up strategy and these adaptations might influence the outputs of the strategy. One way to evaluate the implementation of the strategy is by assessing the implementation fidelity. Implementation fidelity refers to "the degree to which…programs are implemented…as intended by the program developers."[13] This study, therefore, assessed the fidelity of the implementation of the step-based strategy to examine how the SCIROCCO strategy was delivered in practice.

The approach we used for the assessment of implementation fidelity, is based on the work of Carroll et al. [16] and Hasson [17]. The measurement of implementation fidelity is a measurement of adherence, with its subcategories content, frequency, duration and coverage (dose) [16]. Several moderating factors are suggested in the framework to influence the level of fidelity. These factors are: participant responsiveness; program complexity; comprehensiveness of policy description; strategies to facilitate implementation; quality of delivery; recruitment; and context. In this paper, we present findings about the extent to which ''the activities within the SCIROCCO project have been implemented in line with expectations and if, how, and how far relevant initiatives have been developed between 2016 and 2018'' [18]. In this way, factors which facilitated the implementation and factors which were not conducive to the implementation of the strategy are revealed. These insights in the implementation and contextual conditions of the SCIROCCO strategy will provide lessons for those interested in SCIROCCO's step-based approach and future use of its tool and processes to reach progress in the integration of care services. In addition, we aim to contribute to a better understanding on what works in integrated care initiatives and how successful ones can be scaled up.

Methods

<u>SCIROCCO's scaling up strategy</u>

For peer review only - http://bmjopen.bmj.com/site/about/guidelines.xhtml

The SCIROCCO consortium was responsible for the implementation of the strategy. The implementation of the step-based scaling up strategy in the five participating regions was the specific responsibility of the regional partners in each of the five regions. Three regional partners were lead partners for the development of one of the three steps of the scaling up strategy (in collaboration with the other partners within the project). The other partners (referred to as supportive partners) were responsible for the supportive activities within the wider project. These included project coordination, dissemination activities, development of the tool, capturing lessons on the strategy and one independent evaluator (more details are provided in our study protocol). The plan and details on how to implement the step-based strategy were described in the Grant Agreement (protocol). However, not all exact details have been provided as it was indicated that the exact outline and content of several activities were to be developed during implementation. The description of the step-based strategy, as provided in the protocol, is briefly described below.

Step 1: Assessment of maturity requirements in selected GPs

The first step of the SCIROCCO strategy consists of the identification of 30 potentially adoptable GPs in the five regions. Within SCIROCCO good practices are defined as real-life examples of successfully applied innovations in integrated care. The viability of these GPs is assessed using a form which is developed during the implementation. After which 15 GPs are selected as viable scaling up practices. Second, the maturity requirements for transfer of these 15 GPs are assessed using the SCIROCCO tool (see Box 1, all details of the tool are presented in Appendix A), resulting in a guide on how to scale up given the local context in which the practices have been developed. An overview of the three main steps and intended activities of the strategy is provided in Figure 1.

Box 1 Description of the SCIROCCO tool

The SCIROCCO tool consists of 12 dimensions which represent the range of activities that needs to be managed in order to deliver integrated care. The maturity of health care system for integrated care or the maturity requirements of GPs in integrated care are assessed by considering each dimension and allocating a measure of progress or 'maturity' (on a 0-5 scale) to each dimension. The scales include the maturity indicators and reflect the basic indications to look for when assessing the current situation of the maturity of health care system for integrated care or the maturity requirements of GPs. After the assessment, a simple graphical representation (i.e. spider diagram) of status can be derived which reveals areas of strength, further attention and improvement in each of the 12 dimensions.

Step 2: Self-assessment process in the five regions

The second step exists of the development of a guide for the regions describing how to use the tool as a self-assessment tool. Hereafter, the five participating European regions assess their maturity in the adoption for integrated care, using the online SCIROCCO tool, to identify strengths, gaps and areas for improvement. Subsequently, the outcomes of the assessments are collected and analysed, thereby informing about the maturity gaps of a regional health and care system in integrated care. The five regions are then clustered in terms of their complementary strengths and weaknesses. Using the outcomes on clustering of regions, the regions are paired in such a way that the knowledge transfer will flow between the regions with the same strengths (twinning) as well as between the regions scoring high at a particular dimension with the regions scoring low along the same dimension (coaching).

Step 3: Knowledge transfer processes in the five regions

The last step consists of the development of the process for the twinning and coaching activities of five European regions to support the creation of twinning and coaching relationships by using the information (steps 1 and 2 of SCIROCCO's strategy) to construct these relationships. The process of

For peer review only - http://bmjopen.bmj.com/site/about/guidelines.xhtml

knowledge sharing and information flow among the five European regions is facilitated and one twinning and one coaching activity per region is envisaged. The SCIROCCO tool is used to assist in this process. The twinning and coaching activities are organised as face-to-face meetings, webinars and various other online tools.
The last activity comprises the development of an action plan in each of the five European regions. The action plans reflect the findings of the self-assessment process and specifically focuses on addressing the weaknesses in the maturity of regional health and care system. The action plan informs the decision makers about the priority of actions necessary for improvement of their health and care systems. The actual implementation of the plans and monitoring of their progress (i.e. ongoing self-assessment) is not within the scope of the implementation, due to the limited duration of the SCIROCCO project.

Insert: Figure 1 Overview of the three main steps and intended activities per step of the SCIROCCO scaling up strategy

<u>Data collection and study population</u>

To assess the fidelity of each scaling-up step, a multi-method approach was used. Data were collected during the implementation of the strategy from each of the partners and local stakeholders involved in implementation of the strategy. Data collection methods included key informant interviews, focus groups, a questionnaire study and the collection of project documents (i.e. progress reports and deliverables). For the reporting of study, we used the consolidated criteria for reporting qualitative research (COREQ) [19].

The extended conceptual framework for implementation fidelity, developed by Hasson [17] and based on the work of Carroll et al. [16], was used for evaluating implementation fidelity of SCIROCCO's step-based approach (the topics are presented in the next section).

The following topics were leading in the data gathering and -analysis of the data:

- content (the way in which the steps within the strategy were undertaken and changes in these steps);
- frequency and duration (duration was interpreted by checking the timeline and deadlines for the implementation of the three steps);
- facilitating conditions (quality and usefulness of the protocol, guidance and collaboration within the scaling up strategy);
- context (barriers and facilitators in carrying out the strategy);
- participant recruitment procedures;
- points of improvement.

Qualitative data, including semi-structured interviews and working documents (interim report, final report, project deliverables, local reports and e-mails), were the main data sources to explore adherence and possible factors that moderate adherence to the implementation of three steps of the SCIROCCO scaling up strategy. In Table 1 an overview is provided including the examined implementation fidelity components (adherence subcategory and potential moderating factors) in this study, the accompanied research question, data collection procedure/source and planning.

Table 1 Implementation fidelity components (adherence subcategory and potential moderating factors), research question, data collection procedure/source and planning)

Adherence subcategory	Research question(s)	Data collection procedures/sources	Measurement planning

For peer review only - http://bmjopen.bmj.com/site/about/guidelines.xhtml

Content	How are the three steps of the of scaling up strategy delivered in the five regions?	-Semi-structured interview with supportive and regional partners<br><br>-Work documents (i.e. progress reports, interim reports) of supportive and regional partners<br><br>-Deliverables of supportive and regional partners	Alongside SCIROCCO project
Frequency/Duration	How many GP assessments are performed in the five regions?<br><br>How many self-assessments are performed in the five regions?<br><br>How many twinning and coaching activities are performed in the regions?<br><br>What was the duration of the implementation of the three steps?	-Semi-structured interview with supportive and regional partners<br><br>-Work documents (i.e. progress reports, interim reports) of supportive and regional partners<br><br>-Deliverables of with supportive and regional partners	Alongside the SCIROCCO project
Coverage (Reach)	How many local stakeholders per region participated in the different steps of the SCIROCCO strategy?	-Work documents of regional partners<br><br>-Deliverables of regional partners<br><br>-Emails with regional partners	Alongside the SCIROCCO project
Potential moderating factors			
Participant responsiveness	How satisfied were the participants with their participation in the study visits?<br><br>How did the participants perceive the outcomes and relevance of the study visits?	-Focus groups with local stakeholders and regional partners of the five participating regions<br><br>-Short survey with the local stakeholders and regional partners of the five regions on experience in in the study visit	After the study visits
Participant recruitment	What recruitment procedures were used to attract local stakeholders to participate in the three steps of the strategy?	-Semi-structured interview with supportive and regional partners<br><br>-Focus groups with local stakeholders and regional partners of the five participating regions<br><br>-Deliverables with supportive and regional partners	Alongside SCIROCCO project<br><br>After the study visits<br><br>Alongside SCIROCCO project

For peer review only - http://bmjopen.bmj.com/site/about/guidelines.xhtml

Conditions to facilitate implementation	What conditions were used to support the implementation of the SCIROCCO scaling up strategy? How were these conditions perceived by SCIROCCO partners and local stakeholders involved in the strategy?	-Semi-structured interview with supportive and regional partners<br><br>-Focus groups with local stakeholders and regional partners of the five participating regions<br><br>-Work documents (i.e. progress reports, interim reports) of with supportive and regional partners<br><br>-Deliverables of with supportive and regional partners	Alongside SCIROCCO project<br><br>After the study visits<br><br>Alongside SCIROCCO project
Context	What factors at political, economical, organizational and work group level affected the implementation of the scaling up strategy?	-Semi-structured interview with supportive and regional partners<br><br>-Focus groups with local stakeholders and regional partners of the five participating regions<br><br>-Work documents (i.e. progress reports, interim reports) of supportive and regional partners<br><br>-Deliverables of supportive and regional partners	Alongside SCIROCCO project<br><br>After the study visits<br><br>Alongside SCIROCCO project

The semi-structured interviews were conducted over Skype with members of the SCIROCCO project responsible for the different steps in the strategy (n=12). The interviews were held by the first author and lasted about 60 minutes each. Details on the interviews are presented in Appendix B.

Furthermore, five focus groups were organised after each of the five twinning and coaching study visits (on location) and included the regional SCIROCCO partners in the five regions and the external local members who participated in the study visits (referred to as 'local stakeholders'). These local stakeholders were recruited by the regional SCIROCCO project partners. The focus groups were alternately facilitated by the first author and two members of work package (WP8) which was part of the SCIROCCO project and focused on collecting lessons learned on the process of using the SCIROCCO tool and strategy. The three female facilitators possessed a minimum of a master's degree and experience in qualitative research. One of the facilitators holds the position of principal eHealth policy analyst, the other two were researchers working at universities. At the start of the focus groups, the moderators introduced the focus group and themselves, explaining the purpose of the focus group, and requesting the participants to sign the informed consent form (see ethics statement). All participants received an overview of the focus group questions at the beginning of each study visit. Data collected from these focus groups centred on the experiences of the participants on the study visits which were organised as part of step three from the scaling up strategy, and included the following topics: content, participant recruitment procedures, participant responsiveness, and points of improvement. The focus groups lasted approximately an hour. In Table 2 an overview of the characteristics of each focus group is provided. Sometimes the same participants were included in the two focus groups. This happened when participants took part of two study visits. The topics of the focus groups were different. The interviews and focus groups were, after obtaining signed consent, audiotaped and transcribed and during both field notes were made.

After the focus groups were conducted, a survey was distributed among the same participants (n=49) to collect data about participants responsiveness (expectations, satisfaction, clarity and usefulness of results of study visit). The characteristics of the participants who completed the survey are shown in Table 3. Some stakeholders participated in two study visits and completed the survey for both visits.

Patient and public involvement

For peer review only - http://bmjopen.bmj.com/site/about/guidelines.xhtml

One patient representative was involved in the twinning and coaching activities of one site and was involved in the focus group.

Table 2 Characteristics of the focus groups

Location	Subject of focus group	Participants from region (number)	Total number of participant s
1. Puglia	Experience study visit Puglia on GP* in telemonitoring	Experts from Puglia (3), Olomouc (5) and Scotland (6)	14
2. Basque country	Experience study visit Basque country on GP in Advanced Care Planning	Experts from the Basque country (3) and Norrbotten (3)	6
3. Scotland	Experience study visit Scotland on dimension Innovation Management of the SCIROCCO tool	Experts from Scotland (3) and Norrbotten (5)	8
4. Norrbotten	Experience study visit Norrbotten on dimension eHealth & Information services of the SCIROCCO tool	Experts from Norrbotten (4) and Olomouc (4)	8
5. Scotland	Experience study visit on GP in third sector in Scotland	Experts from Scotland (3), Puglia (4) and Basque country (6)	13
		Grand total:	49

*GP= Good Practice

Table 3 Characteristics of participants who completed the survey (n=40)

**Overall**	(n) of participants	
Region of origin participants	Region 1 (Basque Country) = 8<br>Region 2 (Olomouc) = 9<br>Region 3 (Norrbotten) = 10<br>Region 4 (Puglia) = 6<br>Region 5 (Scotland) = 7	
**Per study visit:**		
Puglia (n=12)	Transferring region* Puglia=2	Receiving regions**: Olomouc=5 and Scotland=5
Scotland (n=6)	Transferring region Scotland=2	Receiving region Norrbotten=4
Basque country (n=6)	Transferring region Basque country=3	Receiving region Norrbotten=3
Scotland (n=9)	Transferring region Scotland=0	Receiving region Puglia=4 and Basque Country=5
Norrbotten (n=7)	Transferring region Norrbotten=3	Receiving region Olomouc =4

*The transferring region is the region acting as the “coaching” partner in twinning and coaching activity. **The receiving region, acts as the ‘’learning’’ partner and is the region seeking support from the transferring region to deploy a good practice and/or improve a specific aspect of integrated care.

Data analysis

Data from the interviews, focus groups and documents were analysed using content analysis [20]. A coding scheme, including the implementation fidelity concepts and each intervention component, was used during the coding process. The scheme was tested independently by two researchers (LG,

For peer review only - http://bmjopen.bmj.com/site/about/guidelines.xhtml

HJMV) prior to undertaking the coding process. The analysis of the transcripts and documents was conducted in NVivo 12. The first coder (LG) coded all transcripts using the coding scheme and the second coder (HJMV) operated as a control and coded a random selection of 10% of the transcripts and 10% of the collected documents. The results from this coding process were discussed among the researchers and any disagreement was resolved until consensus was reached. The surveys collected after the twinning and coaching study visits were analysed using descriptive methods in SPSS version 25. Validity of the findings was ensured by using member checks (summaries of the interviews were sent to the respondents, numbers were checked by sending confirmatory emails to the responsible partners) and triangulation (i.e., data from focus groups, documents and interviews were collected to locate/inform the concepts of implementation fidelity) [21], this also ensured data saturation [22].

Results

In this section, first, the results concerning adherence to the three steps of the scaling up strategy during its implementation in the five regions are presented. Here after, the moderating factors which were found to influence the implementation of the scaling up strategy in the five regions are described.

Adherence subcategories

Content

All the steps of the scaling up strategy (maturity requirements in selected GPs, self-assessment process, and twinning and coaching) were implemented with acceptable fidelity. Also, the methodologies developed during the implementation of the steps, which described how to implement the activities in the regions were followed by the regions to a large extent. More details on the content and any deviations observed during the implementation of the steps in the five regions are described below.

Step 1: Maturity assessment of GPs

All the five regions collected data on GPs in their region, implemented the viability assessments of selected GPs and assessed the maturity requirements of the prioritised GPs by applying the tool. In order to evaluate the viability for scaling-up of the SCIROCCO GPs, a six-criterion assessment framework was built based on the criteria used in the European Innovation Partnership on Active and Healthy Ageing repository [23]. The six criteria included: 1. Time needed for the practice to be deployed; 2. Investment per citizen/service user/patient (referring to marginal cost over previous situation);3. Evidence behind the practice;4. Maturity of the practice;5. Estimated time of impact of the practice and 6. Level of transferability of the practice. A deviation was found in delivering the maturity requirement assessment of the selected GPs in the regions, this assessment was performed twice. This was due to observed heterogeneous outcomes across the five SCIROCCO regions. Also, CHAFEA, the fund holder on behalf of Horizon 2020, requested for performing an extra assessment. In Figure 2, the process of the revised assessment is presented, and all the five regions followed the steps of the revised process in their region. The main difference between the first and second assessment was the number of assessors and focus of the assessment. In the first assessment, the focus was on the maturity of the context wherein the GP was developed and was performed by a single representative of the GP. In the second assessment, a group of experts assessed the GP with the focus on the maturity needed to implement the GP in different health and social care settings.

Insert: Figure 2 Revised process on the maturity assessment of Good Practices (GPs)

Step 2: Self-assessment in five regions

The five participating regions implemented the self-assessment process according to the developed methodology (as shown in Figure 3) and applied the tool. All regions wrote a report on the implemented local self-assessment process in their region, including an analysis on the strengths and weaknesses and priority actions for their region.

Insert: Figure 3 Developed methodology on the maturity assessment of health care systems

For peer review only - http://bmjopen.bmj.com/site/about/guidelines.xhtml

Box 2 Description of the Good Practice of one site involved as transferring region in the twinning and coaching activities

Hospital@Home Good Practice is designed as a technological support for already structured activities in home care in Puglia (Italy). The main objective is to reduce hospitalisation and re-hospitalisation and to improve the quality of care for patients at home. In addition, the objective is to:

-Reduce the number of patients with heart disease, diabetes and other chronic diseases in the process of instability

-Activate protected de-hospitalisation

-Optimise the therapy and diagnosis according to international guidelines.

The aim is to implement a new type of telemonitoring, based on continuous collaboration and patient monitoring by different professionals and different users. Patients are telemonitored by their General Practitioners by using the innovative home and health monitoring technological solution (H&H Hospital@Home). This solution is able to detect the main clinical and instrumental parameters in addition to the therapeutic administration, based on oxygen and bronco-aspiration. It is allocated at the patients' home and it is permanently interconnected with the General Practitioner and/or Specialist, by computer, telephone, tablet and other devices.

At the same time, there is a central monitoring room at the hospital for all patients and all devices located at their home. All clinical parameters of patients are stored on a dedicated server, respecting the rules for the respect of privacy. The system allows the healthcare professionals (neurologists, pulmonologist, cardiologists, diabetologists, etc) to monitor and speak with patients remotely. The patients can also activate the visit of the healthcare professionals in their homes. In addition to real-time monitoring of physiological parameters, the healthcare professionals can also monitor the physical and technical characteristics of home device. As a result, it is possible to deliver therapy to the patient remotely.

Step 3 Twinning and coaching

Each of the five SCIROCCO regions were involved in the twinning and coaching processes. The process designed to conduct twinning and coaching activities in the regions consisted of three key phases:

Phase 1: Planning for the twinning and coaching;
Phase 2: Knowledge transfer activities;
Phase 3: Capturing the outcomes of twinning and coaching.

The twinning and coaching activities took place between a transferring region and receiving region. The transferring region was the region acting as the "coaching" partner in twinning and coaching activity. The receiving region was the region seeking support and know-how in order to deploy a GP and/or improve a specific aspect of integrated care and acted as the "learning" partner. During implementation of this step, a variation was observed as one region did not play the role of coaching region due to low maturity scoring across all SCIROCCO dimensions. As a result, one region acted twice as transferring region. Another variation was found in the fact that two regions participated twice as receiving region. A description of the GP of one site involved as transferring region in the twinning and coaching activity is provided in Box 2. Descriptions of all 5 twinning and coaching sites are outlined elsewhere [24].

In Figure 4, an overview of the process of phase 2 on the knowledge transfer activity is provided. Local variation in the implementation of these steps per twinning and coaching activity were observed. This was because the individual regions were provided the opportunity to reflect their local needs and strategic priorities for integrated care in (the scope of) the twinning and coaching process. The implementation of prior contact between the transferring and receiving regions varied (step 3.3). In addition, some of the documentation which were prepared for the twinning and coaching activity were translated. The outline of the program for the study visits also varied per activity (step 3.4) as the different study visits were adapted to the topic of the visit and the needs of the receiving regions. The general outline of the program included presentations of the transferring and/or receiving regions, live demonstrations/site visit of the GP (if relevant) and a facilitated discussion on the maturity requirements using the SCIROCCO tool. During the several study visits, the tool was applied in

For peer review only - http://bmjopen.bmj.com/site/about/guidelines.xhtml

different ways because the partners were interested to test the use of the tool in the twinning and coaching activity. Furthermore, the tool was not explicitly used in one study visit because of time constraints.

With regard to the last activity, developing the action plans (phase 3), the developed methodology included the following steps: the receiving regions were asked to organise a local meeting in their region reflect on the outcomes of knowledge transfer activities and agree on the local priority actions for the transferability of learning. Here after, the action plans were co-designed by transferring and receiving regions. All five regions participated as receiving regions and wrote an action plan reflecting the outcomes of the twinning and coaching activities. Once completed, the intent was to upload the action plans in the online SCIROCCO tool and share these with all relevant stakeholders. In the last step, the plan was to promote the outcomes of the knowledge transfer activities locally and across the regions, no specific details could be retrieved on the last two intended activities.

Insert: Figure 4 Twinning & coaching process

Frequency and duration

In Table 4, a general overview is provided on the frequency of the three SCIROCCO steps, to see whether the target which were set for the different steps were met during the implementation of the strategy. The target number of activities were fully executed during implementation, sometimes even exceeded the target. When looking into the revised maturity assessment of GPs (step 2), one deviation was observed during the second GP assessment of one GP in Region 5. During the time of the second assessment the GP was embedded as part of the routine practice at national level, therefore, it was not possible to conduct a second assessment on the GP itself.

Table 4 Frequency of SCIROCCO's activities (figures represent number unless indicated otherwise)

Step	**Activities**		**Project target**	**Delivered**	**Region 1(B)**	**Region 2 (O)**	**Region 3 (N)**	**Region 4 (P)**	**Region 5 (S)**
1	Number of viability assessment of GPs in the five regions		30	32 (106.7%)	7	4	6	8	7
	Number of GPs selected and assessed for maturity assessment	Assessed with first designed methodology	15	15 (115.4%)	3	3	3	3	3
		Assessed with revised methodology (including all steps conducted of the revised process)	15	14 (107.7%)	3	3	3	3	2
2	Regions performed the complete self-assessment process		5	5 (100%)	1	1	1	1	1
	Completed and documented assessments		5	5 (100%)	1	1	1	1	1
3	Number of regions which performed role as transferring/ coaching region in twinning and coaching activity		≥5	5 (100%)	1	0	1	1	2
	Duration of study visit (up to two days)				1,5 day	0 day	1,5 day	1,5 day	One 1,5 day and one 1 day
	Number of regions which performed the role as receiving region		≥5	7 (140%)	1	2	2	1	1

For peer review only - http://bmjopen.bmj.com/site/about/guidelines.xhtml

	Agreed action plans to transfer and/or scale-up interventions		≥5	7 (140%)	1	2	2	1	1

During the implementation of the steps, adjustments were made in the timeline of all the steps. An overview of the original timeline and the executed timeline is provided in Appendix C. A delay was observed in organising the local self-assessment processes in the regions (step 2) since the engagement of the local stakeholders for the self-assessment took much longer than anticipated. In addition, after the self-assessment process, the re-assessment of the maturity requirements of 15 GPs (step 1) needed to be executed in the five regions. Since the planning of the implementation of step 3 was dependent on the outcomes of steps 1 and 2, step 3 was organised with a delay of 6 months. The parallel development of SCIROCCO methodology for twinning and coaching allowed the immediate start of the approach in the regions, including the development of regional action plans. In the original program, when the partners were thinking about the description of the steps, what might have been improved was the duration of the strategy which was indicated by one supportive partner.

Coverage

The coverage of the local stakeholders participating in the three main steps of the implemented strategy are shown in Table 5. Since the methodology of the different activities was designed during implementation, the target number for identifying the local stakeholders to participate in the different activities per region was not defined beforehand but were indicated during implementation. These target numbers for the different activities are also presented in Table 5.

For the revised assessment of step 1, the regional SCIORCCO partners were instructed to identify experts with different profiles, backgrounds and experiences from both managerial and practice profile. The regions adapted the identification of experts to their local context/local GPs, which lead to variation in the structure and the number of stakeholders involved per region. For the activities of step 2 and 3, a variation in structure and the size of the local teams of engaged experts per region was also observed. The local variations were the results of the fact that for step 2, the scope of the self-assessment activity was defined by each individual region reflecting the structure of their health care system and the concept of integrated care and the regions were instructed to capture a diversity of perspectives in the assessment process, including different disciplines, sectors and positions in the organisation. For step 3, the scope of the twinning and coaching process was also defined by each individual region reflecting the local need and strategic priorities for integrated care and the regional partners selected the stakeholders based on their experiences with the subject of the twinning and coaching activities. This also resulted in variations in the local teams of engaged experts per region.

Table 5 Coverage of local stakeholders per step of the scaling up strategy

**Step**	**Activities**	**Region 1 (Basque Country)**	**Region 2 (Olomouc)**	**Region 3 (Norrbotten)**	**Region 4 (Puglia)**	**Region 5 (Scotland)**	**Total**
1	Number of participants assessed the maturity of the context where the GPs* was developed (first methodology)	3x1	3x1	3x1	3x1	3x1	15
	Number of individual questionnaires collected per GP (4-8 experts maximum) (second methodology)	1x4<br>1x4<br>1x4	1x2<br>1x3<br>1x3	1x5<br>1x4<br>1x4	1x5<br>1x5<br>1x6	1x3<br>1x4	56
	Number of participants in face-to-face workshop per GP (second methodology)	1x4<br>1x4<br>1x4	1x2<br>1x3<br>1x3	1x5<br>1x4<br>1x4	1x5<br>1x5<br>1x6	1x3<br>1x4	56
2	Number of stakeholders invited to fill in the	10	>20	9	11	12	

For peer review only

	questionnaire of the SCIROCCO tool						
	Number of individual questionnaires completed (target max. 10 local stakeholders per region)	10	5	7	11	9	42
	Number of participants in face-to-face workshop	9	5	7	11	5	37
3		Study visit Basque Country (GP)	Study visit Norrbotten (dimension)	Study visit Puglia (GP)	Study visit Scotland (GP)	Study visit Scotland (dimension)	
	Number of actively involved participants in the study visit (transferring and receiving region)	15	19	22	14	15	85
	Number of actively involved participants from the receiving region in the study visit (max. 5 per region)	5 (one left early)	4	5 (Olomouc)<br>6 (Scotland)	6 (Basque country)<br>4 (Puglia)	4	34

*GP= Good practice

<u>Moderating factors</u>

Participant recruitment

Recruitment for the GP assessment (Step 1)

The regional SCIROCCO partners were provided instructions on how to perform the assessments of the GPs in their region and how to select and invite the local stakeholders (GP leaders). The recruitment process for the first assessment of the GPs was implemented in all the five regions in line with the instructions provided. The regional SCIROCCO partners invited the practice leaders to participate in the GP assessment, and they received guidelines which described the steps to follow.

In the second assessment, using the revised methodology, the GP assessment process was changed, and the assessment was performed by a multi-disciplinary team rather than single key informant. Again, the regional SCIROCCO partners were introduced to the revised methodology and received new instructions. A slight variation among the regions was observed in the identification procedure of stakeholders. In four regions the local SCIROCCO project partners identified the local stakeholders themselves (Regions 1, 2, 4 and 5). Another region identified local stakeholders with the support of the local steering group (Region 3), these stakeholders were included in a local project team which participated in various SCIROCCO activities. All the regional SCIROCCO project partners sent an invitation via e-mail to all the identified local stakeholders in the five regions and all experts were invited to use the online version of the SCIROCCO tool to conduct their individual assessments. A slight variation was observed as one of the regions organised a meeting to introduce the project to the stakeholders and explain the assessment process (Region 1).

Recruitment for self-assessment process (Step 2)

All the regional SCIROCCO partners received instructions on how to perform the self-assessment in their regions and how to recruit the local stakeholders including predefined selection criteria. Despite the provided instructions and predefined invitation letter, the regional SCIROCCO partners needed to provide additional efforts to explain to the local stakeholders how to participate in the self-assessment process. As a result, extra informative documents and illustrative videos on how to use the SCIROCCO tool in the self-assessment process were provided. Furthermore, the tool was translated in three additional languages. In the regions different procedures to identify the local experts in the self-assessment process were implemented. In four regions the regional SCIROCCO partners recruited the local experts to participate in the self-assessment activities (Region 1, 2, 4 and 5). Another regional partner identified local stakeholders with the support of the local steering group

For peer review only

For peer review only - http://bmjopen.bmj.com/site/about/guidelines.xhtml

(Region 3). In addition, the recruitment in the regions varied in using different communication methods and in the length of the process. In some regions, there was a need to clearly communicate the added-value and benefits of the assessment process (Regions 1 and 5 and organised a prior meeting to explain the self-assessment process. One region (Region 4) translated the prepared documents. Another region, called the identified experts by phone (Region 2). In this region, some of the stakeholders expressed that it was hard to respond to the tasks (filling in the on-line tool) as the concept of integrated care is not an urgent topic on the agenda of their country. Subsequently, the recruitment of local stakeholders was a difficult and long procedure for this region. Finally, in some regions it was a challenge for the local experts to reserve time to participate in the activities (Regions 2 and 3).

Recruitment for twinning and coaching (Step 3)

The local experts who participated in the twinning and coaching activities were identified by the regional SCIROCCO partners in all five regions. The recruitment of experts varied per region, in terms of use of different communication channels and in length of the process. Exact details on how the experts were recruited could not be retrieved from the data. A few experts were already involved in previous activities within the SCIROCCO project and most experts were new to the project.

Participant responsiveness

The majority stakeholders were observed to have good rapport with the study visits. Most stakeholders who participated in the study visits indicated that the information provided on the content and process prior to the study visit was clear. Also, most stakeholders agreed that they could ask and discuss what they wanted during the visits and the visits matched with the expectations of most of them. Lastly, the majority of the experts indicated that the content of the study visit should somewhat or moderately influenced decisions in their region (see Table 6 for all details).

Table 6 Experiences of stakeholders about study visit (n=40) (figures in %)

**Question**	**Answer categories**				
	Very unclear	Unclear	Neither clear, nor unclear	Clear	Very clear
Q1. Prior to the study visit, how clear was the information provided on the content and process of the study visit?	5	0	5	55	35
	Strongly disagree	Disagree	Undecided	Agree	Strongly agree
Q2. Were you able to ask and discuss everything you wanted during the study visit?	0	7.5	10	35	47.5
	Much less than expected	Less than expected	As expected	More than expected	Much more than expected
Q3. How well did the study visit matches your expectations?	0	10	22.5	35	32.5
	Not at all influence	Slightly influence	Somewhat influence	Moderately influence	Extremely influence
Q4. To what extent do you think the content discussed during the study visit should influence decisions in your region? (1 answer missing)	0	7.5	42.5	40.5	7.5

Most stakeholders indicated appreciation for the usefulness of the study (explicitly addressed in 4 out of 5 study visits). The sharing of experiences and collaboration during the study visits were explicitly mentioned in two focus groups. A participant mentioned in focus group 4 (FG4) that *''we think you have something you can share with us and something we can learn from you.''* The study visits were considered as an inspiration and regarded as an *''injection of optimism''* (FG1) to improve elements in

For peer review only - http://bmjopen.bmj.com/site/about/guidelines.xhtml

their own regions. In particular, the onsite experiences were appreciated in three study visits, were practices were visited *''in their real context''*(FG 2). However, in another study visit a respondent indicated that the visit was a bit less concrete and practical than expected.

The scheduled amount of time for the study visits was in some cases regarded as sufficient and in other cases this was perceived as insufficient. Some respondents indicated that they did not have enough time to reflect on information which was shared. Another respondent mentioned that prior to the visits, it would be useful to go through the tool and have a meeting or webinar with the transferring regions to have a better understanding of what will be the interest of the visiting regions. When two regions participated as receiving regions in the study visit, a few stakeholders indicated that more time would probably be needed for the study visit. On the site of the study visit organisers (transferring regions) it was indicated in two focus groups that the organisations of the visit might have benefited from more time to prepare, to for example arrange ''even more practical examples'' (FG4) and to be able to *''to have the perfect team in place''*(FG3).

During the study visit, the tool was tested in facilitating the discussion between the regions. The use of the tool was regarded in four study visits as a support to facilitate the discussion by providing a structure to the conversation. Not all stakeholders had experience using the SCIROCCO tool in the study visits. Two respondents explicitly mentioned that they thought the tool was difficult to understand the first time. A difficulty experienced with the tool was mentioned in the language of the tool, as it was not regarded as the simplest English. According to a stakeholder the terms used in the tool need to be locally interpreted, *''as it should be translated into a local terminology and context''* (FG3). Furthermore, it was indicated by a few stakeholders that having a translator present during the study visit was critical.

Conditions to facilitate implementation

Project coordination, effective communication and guidelines

The central (strategy) coordination was regarded to be a supportive factor to implement the steps of the strategy in the five regions (according to all partners). Since the start of the strategy, bi-weekly telecom conferences (virtual meetings) took place over the course of the entire implementation period to facilitate effective communication with the partners. During these telecoms, among other things, the draft methodologies designed for the scaling up steps were discussed. Several additional online meetings within the consortium were organised to make sure that all partners understood how to implement the different steps in their region. In addition, five project assembly meetings were organised were the partners were given the opportunity to share and discuss their work on implementing the activities in the five regions. Furthermore, written guidelines on how to implement the different activities in the regions were also shared by email to the regional partners. In addition, three sets of detailed guides were designed to guide the use of the online SCIROCCO tool. The full set of instructions was also included directly in the online version of SCIROCCO tool. In addition, several supportive training materials and demo videos were developed and could be accessed online. The available methodology was regarded to be helpful in providing consistency in the self-assessment processes and the twinning and coaching process.

Flexible approach

The flexible approach within the strategy to develop the exact design of the steps of the strategy during the implementation and the opportunity to sometimes deviate from the original plan was regarded as a facilitator for implementation as indicated by the regional partners of two regions. One regional partner in interview 7 (I7) indicated: *''you give kind of an idea on how it should work, but then in real life things happen in a different way.''* One regional partner indicated that the flexible/open approach for developing the steps could have influenced the planning of the strategy, because in-depth discussions among the consortium partners or the representatives of regions sometimes took more time than anticipated.

Supportive attitude of regional partners

The exact design of the steps of the strategy were developed in collaboration with the support of other

For peer review only - http://bmjopen.bmj.com/site/about/guidelines.xhtml

work packages and the project coordination. The collaboration among the different regional and supportive partners was regarded as open and a positive experience which was explicitly mentioned by four partners. A facilitator for implementation mentioned by two regional partners was regarded in the commitment of all the project partners to achieve the objectives of the project. Also, one partner further indicated that the flexible attitude of all project partners was regarded to facilitate implementation.

Regional conditions

The regional partners of two regions indicated that they had several co-workers from their region involved in the project which supported the implementation of the project activities in their region. For example, Region 3 had set-up a local steering group, which appointed people to be part of a local project team, including people with different roles and responsibilities. This local working group worked on the activities of the SCIROCCO project and from this group some stakeholders were also recruited to participate as the stakeholder team throughout the project.
Lastly, before step 2 was implemented in all the five regions, the designed process was pilot tested in one region. One regional partner expressed that this was regarded as facilitative for the implementation in the other regions. For the implementation of the GP assessment (step 1) the regional partner of another region indicated that it perhaps would have been good to also pilot test the method in their own region before other regions followed.

Context

The different regional contexts of the five SCIROCCO regions were found to influence the implementation of the steps in the five regions. Several context factors may have moderated adherence to the various scaling up steps.

Language and conceptual adaptations on integrated care

During implementation, an unanticipated activity was the translation of the tool to improve stakeholders' experience with the tool. In addition, the provided invitation letters needed to be translated and all the individual assessments (for step 1 and 2) as well as consensus-building workshops were held in local languages except for one region which was also held the workshop in English (Region 3). During the implementation of the project it was observed that the translation of the tool is not enough. To improve the understanding of the tool, it was indicated that the concepts of the tool needed to be adjusted to local linguistic and contextual aspects of integrated care. However, this cross-cultural adaptation was found not to be feasible in the duration of the strategy and the recommendation was that this should be considered as a potential improvement of the tool in the future.

Level of development in integrated care

The variation of the regions in the level of development and implementation of IC influenced the implementation. During the implementation of the SCIROCCO steps 2 and 3, the five regions were able to define the scope for the self-assessment process and the twinning and coaching activities to reflect the structure of their healthcare systems, the concept of integrated care (step 2) and the local needs and strategic priorities for integrated care (step 3). In one region, Region 2, the concept of integrated of care was relatively new. For this region, IC is not widely known, and stakeholders have limited knowledge about nor experience with this approach. Hence, the assessment processes proved to be a complex task for the region, especially in the engagement with local stakeholders. Also, the region did not play the role of transferring region due to low maturity scoring across all SCIROCCO dimensions. Furthermore, it was indicated by one partner that in the regions where the IC agenda was strongly established, the implementation of the activities of the project ran smoother.

Local perspectives

The variation in implementing the steps may have also been influenced by the different perspectives of the SCIROCCO partners in the regions. One supportive partner mentioned that the regions varied in their perspectives as some were more policy driven, some more practical or research focused, which

For peer review only - http://bmjopen.bmj.com/site/about/guidelines.xhtml

could have influenced the implementation, since the implementation of step 2 and 3 were adapted to the regional context and needs.

Changes in local environment

During implementation of SCIROCCO, changes in the local environment of the different participating regions were indicated to possibly influence implementation. Some organisational changes occurred in the public authorities were the regional partners were affiliated. For one region the focus of their health system agenda changed. In another region, the change in management of their organisation influenced how well known the project was and how much leverage the project had. Another regional change during implementation was observed on the level of a GP. One GP was embedded as part of the routine practice at national level. As such, this GP could not be included in the second assessment on the GP in step 1.

Discussion

This study assessed the implementation fidelity of a step-based scaling up strategy for IC which was implemented in five European regions as part of a cross national project. The results show that all steps of the scaling up strategy were implemented with acceptable fidelity. All five regions conducted the GP self-assessments (step 1) and undertook the self-assessment process of their health care system context in their regions (step 2). Each region also participated in the twinning and coaching process and wrote one or two action plans (step 3). In addition, the targets which were set for each step were met or even exceeded (frequency). However, some deviation was also found. First, the GP assessments in the regions were implemented twice (step 1) and one region did not participate as transferring region in the twinning and coaching activity (step 3). Also, the duration of the steps 1 and 2 took longer than anticipated. Lastly, the coverage of the local experts per region who participated in the three scaling up steps varied in structure and size.

We found several factors that influenced implementation fidelity of the scaling up strategy. These included facilitating conditions in the flexible approach and coordination, positive participant responsiveness, regional variability in participant recruitment procedures and several contextual factors. Supportive influencing factors for implementation of the three steps in the five regions were found in good coordination to implement the steps, the use of effective communication strategies among the partners, the provision of guidelines on how to implement the processes and the SCIROCCO tool in the regions. Furthermore, the flexible approach, the supportive attitude of partners, and certain regional conditions (i.e. having a local steering group and regional pilot testing) have facilitated the implementation of the steps in the regions. A last important factor was the positive experience of the local stakeholders which participated in the study visits.

Several factors found in facilitating conditions (the flexible approach), the local variations and adaptions in participant recruitment procedures and several contextual factors moderated adherence to the implementation of the strategy. The contextual factors which influenced the implementation of the strategy were reflected in the local differences in language and conceptual understanding of integrated care among the regions. In addition, the different health care system contexts, level of development in IC, and the local needs and strategic priorities resulted in variations in implementation of the strategy. Lastly, regional changes during the implementation of the strategy also influenced adherence. For example, for step 2 a selected GP could not be included in the second assessment as, during the lifespan of the strategy, it was embedded as part of the routine practice at the national level.

The implementation of the strategy consisted of a partly standardised and partly flexible approach. Flexible interventions present a challenge for evaluators, as flexibility and fidelity can be at odds with each other. The flexible approach to adapt or deviate from protocols is in contrast with examining the implementation fidelity of an intervention to its protocol. The discussions about the conflict on how to consider fidelity and the extent to which adaptation across contexts is fair or needed are unresolved [25]. However, adapting health service delivery to local conditions has several advantages. The variety in implementation found in our study corresponds to the recognition by Carroll et al., that an ''intervention cannot always be implemented fully in the real world. Local conditions may require it to be flexible and adaptable [16].'' Furthermore, it has the potential to improve program effectiveness by

For peer review only - http://bmjopen.bmj.com/site/about/guidelines.xhtml

means of increased program feasibility and increased user adoption [16]. In such a case, it is important to take into account flexibility during implementation because of needed adaption to the local conditions. This occurred within SCIROCCO as the scope of the activities were informed by each individual region reflecting the structure of their healthcare systems, the framework of IC and the local need and strategic priorities for integrated care. To draw valid conclusions about the fidelity of an implementation, it is important to accomplish a good balance between adherence to a protocol on the one hand and the required flexibility for adaptations on the other hand.

To our knowledge, this study is the first to assess the fidelity of the implementation of an international scaling up strategy for integrated care. Most studies which assess the implementation fidelity focus on the implementation of national (complex health) interventions or programs [26–28]. Consequently, we were not able to compare our findings to similar studies. Notwithstanding, our study shows that assessing the implementation of an international step-based scaling up strategy by using the modified version of the Conceptual Framework for Implementation Fidelity [17] is feasible. Hence, we encourage others who work or plan to work on similar strategies to study the implementation fidelity of such strategies, to clarify the factors that are associated with the implementation of their strategy.

Nolte et al. explored the national experiences with developing, implementing and impacting the wider system context of integrated care in three countries [29]. They concluded that ‘‘a more formal strategy’’ for expansion is needed, as the wider dissemination of the projects studied occurred in ‘’an incremental and somewhat haphazard way’’[29]. The scaling up strategy presented in this study focuses on the adoption of integrated care initiatives to other country settings, which is challenging because of different contexts. The scaling up strategy provides an encouraging example of a structured approach to scaling up of integrated care initiatives in an international context. The results of this study provide insight in the factors which need to be considered when implementing the strategy.

Strengths and limitations

The opportunity to assess the implementation of the SCIROCCO strategy in its real-life setting of five European regions enabled us to collect several types of data, to triangulate data, and hence enhance in-depth insight into the implementation. Furthermore, our role within the project provided a unique opportunity to obtain a close collaboration with the partners to collect the data needed for the implementation fidelity assessment. However, being a partner in the consortium responsible for undertaking the evaluation activities, the subjective experiences of the participants (supportive partners, regional partners and local stakeholders) could have been influenced by social-desirability and/or recall bias.

We were aware of our role and to safeguard objectivity we did not interfere with implementation of the project. However, due to time constraints and a high demand on the participants, we were not able to collect data on all pre-planned moments which limited the study coverage of the potential factors influencing the implementation of complex interventions. Only data from one regional partner could be collected during implementation, whereas the rest of the data were collected at the end of the implementation. This limited our insight into the experiences and involvement of participants in other steps. To be able to explain ratings more in-depth we recommend future researchers to examine participant experiences at several moments during the process of implementation. In addition, we have no specific information about the non-responders of the focus groups, since local stakeholders were recruited by the regional SCIROCCO partners we did not have insight in how many local stakeholders were approached by the partners. Therefore, we were unable to track the response rate in the study. Several factors may have contributed to non-responses, which could be the time demands on some respondents of participating in the SCIROCCO project itself (it took at least two full days and traveling time) or not seeing the immediate benefit from participating.

Due to the nature of the strategy, where the methodologies of the steps were developed during the implementation (including identification of the number of stakeholders), measurement of adherence was challenging. This corresponds to other studies who also explored the implementation fidelity with a lack of defined points of references for assessment [26,27,30]. The opportunity to work closely with program implementers ensured us to be kept up to date on the changes regarding revisiting reference points with the evolution of the strategy.

Conclusion

For peer review only

For peer review only - http://bmjopen.bmj.com/site/about/guidelines.xhtml

Overall, the SCIROCCO strategy was regarded an encouraging approach offering regions a tailored but flexible path intended to facilitate progress in integrated care. The multi-method design of this study has yielded knowledge about what elements are involved in implementing a European scaling up strategy concerning IC initiatives. The use of a theoretical framework helped us to document the process by which the strategy was implemented and understand the level of implementation fidelity achieved. The insights obtained could support other regions, not being part of the SCIROCCO project, on what to consider when interested to use the tool and processes to achieve knowledge transfer with other regions to ultimately scale-up IC initiatives. Implementing the strategy might be challenging for regions which are less known to concept of integrated care and when the strategy gets modified depending on the local context of regions. Furthermore, the implementation of the steps in the five regions and this evaluation study took place within the boundaries of a project, where dedicated resources were provided. An important question is whether the SCIROCCO strategy will be as successful without these available resources and whether the evaluation study will encounter other factors that apply to the implementation in these settings. To test the external validity of study findings, it would be recommended to also test the SCIROCCO strategy in other countries both in- and outside Europe. Furthermore, lessons on implementation of the developed action plans would also be useful for other interested regions.

Footnotes

Abbreviations

IC: integrated care, GP: good practice, SCIROCCO: Scaling Integrated Care in Context.

Ethics approval and consent to participate

The conduct of this study was granted permission by the Institutional Review Board of the Vrije Universiteit Brussel (reference: B.U.N. 143201734384). Participant consent was requested before the interviews and focus groups in the form of an informed consent. Signed written consent forms were completed by all participants.

Consent for publication

Not applicable.

Availability of data and material

The datasets used and analysed during the current study are available from the corresponding author on reasonable request.

Competing interests

The authors declare that they have no competing interests.

Funding

This study was undertaken within the Scirocco project which was co-funded by the Health Programme of the European Union under Grant Agreement 710033 (CHAFEA). The funding body was neither involved in the design of the study nor the collection, analysis, and interpretation of data and writing of the manuscript.

Authors' contributions

LG and HJMV provided valuable contributions to the design of the work and provided substantial contributions to the analysis of the work. Both authors provided significant contributions to the acquisition of data, interpretation of data and writing of the manuscript. INF and DD reviewed the manuscript critically and substantively revised the work. All authors read and approved the final manuscript.

Acknowledgements

We would like to thank all SCIROCCO project partners and local experts for their time and valuable contribution in the study.

Patient and public involvement

One patient representative was involved in the twinning and coaching activities of one site and was involved in the focus group.

Reference

For peer review only - http://bmjopen.bmj.com/site/about/guidelines.xhtml

1 Gröne O, Garcia-Barbero M. Integrated care: a position paper of the WHO European Office for Integrated Health Care Services. *Int J Integr Care* 2001;**1**:e21.

2 Amelung V, Stein V, Goodwin N, *et al. Handbook Integrated Care*. Springer International Publishing 2017. doi:10.1007/978-3-319-56103-5

3 Nolte E, Pitchforth E. What is the evidence on the economic impacts of integrated care? 2014.

4 Bautista MAC, Nurjono M, Lim YW, *et al.* Instruments Measuring Integrated Care: A Systematic Review of Measurement Properties. *Milbank Q* 2016;**94**:862–917. doi:10.1111/1468-0009.12233

5 Busetto L. *Great Expectations: The Implementation of Integrated Care and Its Contribution to Improved Outcomes for People with Chronic Conditions*. Int. J. Integr. Care. 2017;**16**. doi:10.5334/ijic.2555

6 Goodwin N. Improving Integrated Care: Can Implementation Science Unlock the 'Black Box' of Complexities? *Int J Integr Care* 2019;**19**. doi:10.5334/ijic.4724

7 Hunter A, Brewer J. Designing Multimethod Research. In: Hesse-Bibe S, Johnson R, eds. *The Oxford Handbook of Multimethod and Mixed Methods Research Inquiry*. London: : Oxford University Press 2016. 85–205.

8 Chambers DA, Glasgow RE, Stange KC. The dynamic sustainability framework: Addressing the paradox of sustainment amid ongoing change. *Implement Sci* 2013;**8**:117. doi:10.1186/1748-5908-8-117

9 Lamont T, Barber N, Pury J de, *et al.* New approaches to evaluating complex health and care systems. *BMJ* 2016;**154**:i154. doi:10.1136/bmj.i154

10 Drabble SJ, O'Cathain A. Moving from Randomized Controlled Trials to Mixed Methods Intervention Evaluation. In: *The Oxford Handbook of Multimethod and Mixed Methods Research Inquiry*. London: : Oxford University Press 2015. 406–2.

11 Berwick DM. The Science of Improvement. *JAMA* 2008;**299**:1182.

12 Henderson D, Pavlickova A, Lewis L. Scalability and transferability of good practices in Europe: What does it take? *Int J Integr Care* 2016;**16**:1–2.

13 Dusenbury L. A review of research on fidelity of implementation: implications for drug abuse prevention in school settings. *Health Educ Res* 2003;**18**:237–56. doi:10.1093/her/18.2.237

14 Mccormack B, Kitson A, Rycroft-malone J. Getting evidence into practice : the meaning of ' context '. *J Adv Nurs* 2001;:94–104.

15 Greenhalgh T, Robert G, Bate P, *et al.* How to spread good ideas: A systematic review of the literature on diffusion, dissemination and sustainability in health service delivery and organisation. *Milbank Q* 2004;:426. doi:10.1111/j.0887-378X.2004.00325.x

16 Carroll C, Patterson M, Wood S, *et al.* A conceptual framework for implementation fidelity. *Implement Sci* 2007;**2**:40. doi:10.1186/1748-5908-2-40

17 Hasson H. Systematic evaluation of implementation fidelity of complex interventions in health and social care. *Implement Sci* 2010;**5**:67. doi:10.1186/1748-5908-5-67

18 Grooten L, Alexandru C-A, Alhambra-Borrás T, *et al.* A scaling-up strategy supporting the expansion of integrated care: a study protocol Article information. *J Intgerated Care* 2019;**27**. doi:10.1108/JICA-04-2018-0029

19 Tong A, Sainsbury P, Craig J. Consolidated criteria for reporting qualitative research (COREQ): A 32-item checklist for interviews and focus groups. *Int J Qual Heal Care* 2007;**19**:349–57. doi:10.1093/intqhc/mzm042

20 Weber RP. *Basic content analysis.* Beverly Hills, CA: : Sage 1990.

21 Creswell JW, Miller DL. in Qualitative Inquiry. *Theory Pract* 2000;**39**:124–30.

For peer review only - http://bmjopen.bmj.com/site/about/guidelines.xhtml

22 Fusch PI, Ness LR. Are We There Yet? Data Saturation in Qualitative Research. *Qual Rep* 2015;**20**:1408–16.

23 European Innovation Partnership on Active and Healthy Ageing, Repository of innovative practices. https://ec.europa.eu/eip/ageing/repository_en

24 Grooten L, Vrijhoef HJM, Alhambra-Borrás T, *et al.* The transfer of knowledge on integrated care among five European regions: a qualitative multi-method study. *BMC Health Serv Res* 2020;**20**:11. doi:10.1186/s12913-019-4865-8

25 Moore G, Audrey S, Barker M, *et al.* Process evaluation of complex interventions. *BMJ* 2015;**350**:h1258.

26 Nurjono M, Shrestha P, Ang IYH, *et al.* Implementation fidelity of a strategy to integrate service delivery: learnings from a transitional care program for individuals with complex needs in Singapore. *BMC Health Serv Res* 2019;**19**. doi:10.1186/s12913-019-3980-x

27 Muntinga ME, Van Leeuwen KM, Schellevis FG, *et al.* From concept to content: assessing the implementation fidelity of a chronic care model for frail, older people who live at home. *BMC Health Serv Res* 2015;**15**:18. doi:10.1186/s12913-014-0662-6

28 Stijnen M, Jansen M, WJ, *et al.* Nurse-led home visitation programme to improve health-related quality of life and reduce disability among potentially frail community-dwelling older people in. *BMC Fam Pract* 2014;:1–14.

29 Nolte E, Frølich A, Hildebrandt H, *et al.* Implementing integrated care: A synthesis of experiences in three European countries. *Int J Care Coord* 2016;**19**:5–19. doi:10.1177/2053434516655626

30 Hasson H, Blomberg S, Duner A. Fidelity and moderating factors in complex interventions: a case study of a continuum of care program for frail elderly people in health and social care. *Implement Sci* 2012;**7**:1–11. doi:Artn 23\rDoi 10.1186/1748-5908-7-23

For peer review only

For peer review only - http://bmjopen.bmj.com/site/about/guidelines.xhtml

For peer review only

For peer review only - http://bmjopen.bmj.com/site/about/guidelines.xhtml

Main scaling up steps of SCIROCCO strategy

**Step**	**1**	**2**	**3**
	Assessment of maturity requirements of GPs in the five regions	Self-assessment process in the five regions	Twinning and coaching (Knowledge transfer) in the five regions
**Objectives/activities**	a) Identify GPs<br>b) Assess viability GPs and select 15 with the potential for scaling-up<br>c) Assess maturity requirements GPs	a) Develop guide for self-assessment<br>b) Self-assessment in five regions<br>c) Collect and analyse data from the self-assessment process to identify strengths and weaknesses of the five European regions<br>d) Clustering of regions in terms of their complementary strengths and weaknesses	a) Develop the process for twinning and coaching activities<br>b) Facilitate process of knowledge sharing and information flow among five European regions using the tool to facilitate this process<br>c) Develop action plan in each of the five European regions

Figure 1 Overview of the three main steps and intended activities per step of the SCIROCCO scaling up strategy

89x89mm (600 x 600 DPI)

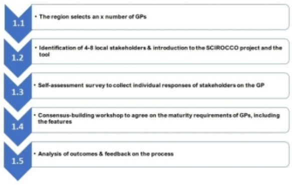

Figure 2 Revised process on the maturity assessment of Good Practices

89x89mm (600 x 600 DPI)

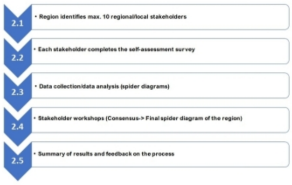

Figure 3 Developed methodology on the maturity assessment of health care systems

89x89mm (600 x 600 DPI)

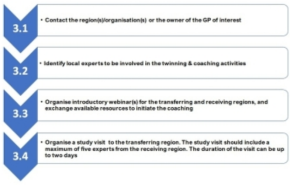

Twinning & coaching process

89x89mm (600 x 600 DPI)

Scirocco
Scaling Integrated Care in Context

Maturity Model for Integrated Care

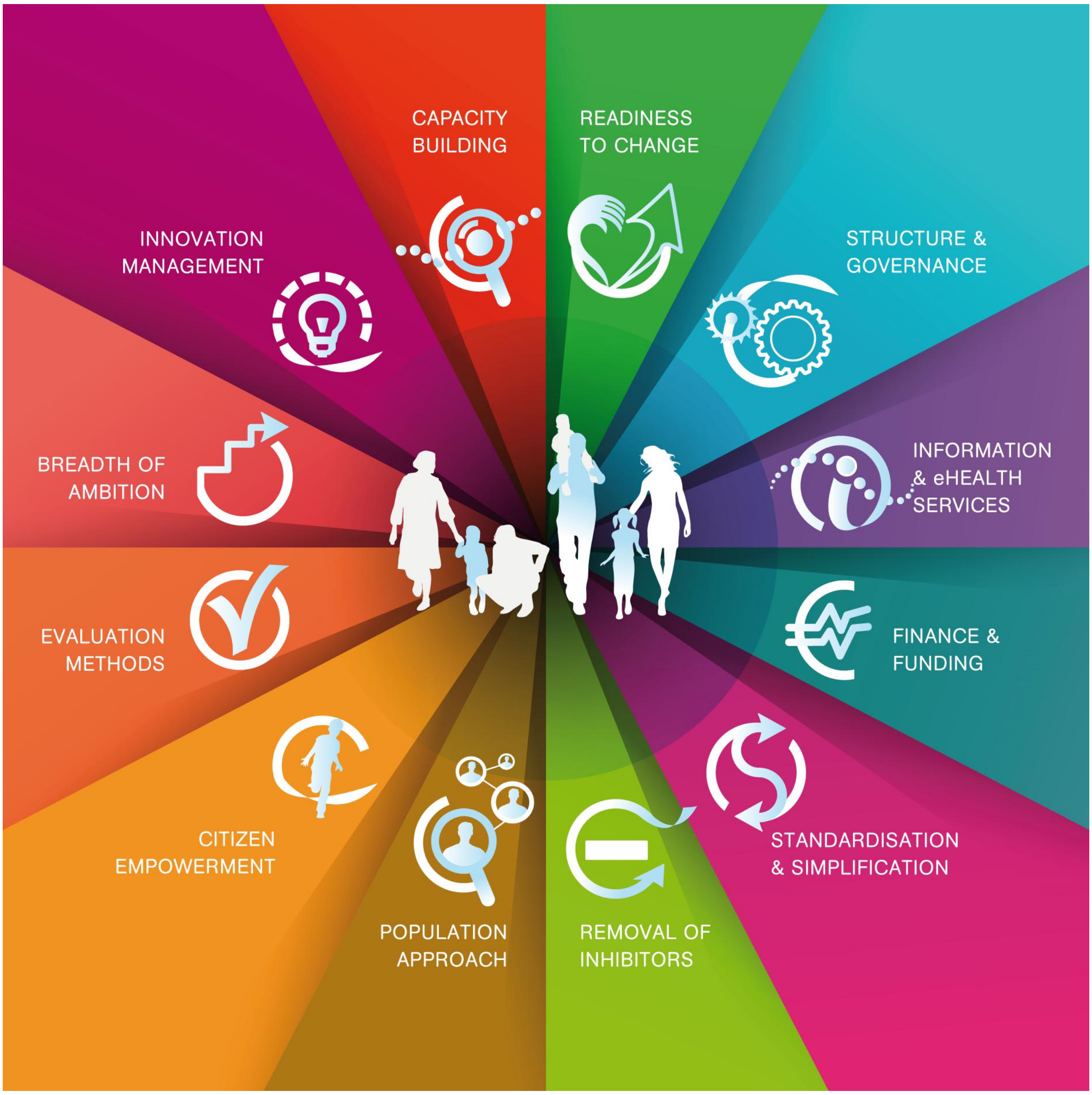

1. Readiness to Change

Objectives:

If the existing systems of care[1] need to be re-designed to provide a more integrated set of services, this will require change across many levels, the creation of new roles, processes and working practices, and new systems to support information sharing and collaboration across care teams. This will be disruptive and may be viewed negatively by workers, press and public, so a clear case needs to be made for those changes, including a justification, a strategic plan, and a vision of better care.

- Creating a compelling vision, with a real sense of urgency, and enlisting stakeholder support including political leadership, management, care professionals, public and press.
- Accepting the reality that care systems are unsustainable and need to change.
- Taking into account the need to address the risk of health inequities.
- Publishing a clear description of the issues, the choices that need to be made, and the desired future state of the care systems, stating what will be the future experience of care.
- Creating a sense of urgency to ensure sustained focus, and building a 'guiding coalition' for change.

Assessment scale:

0 – No acknowledgement of compelling need to change.

1 – Compelling need is recognised, but no clear vision or strategic plan.

2 – Dialogue and consensus-building underway; plan being developed.

3 – Vision or plan embedded in policy; leaders and champions emerging.

4 – Leadership, vision and plan clear to the general public; pressure for change

5 – Political consensus; public support; visible stakeholder engagement.

2. Structure & Governance

Objectives:

The broad set of changes needed to deliver integrated care at a regional or national level presents a significant challenge. It needs multi-year programmes with efficient change management, funding

---

[1] The term care refers to both health and social care.

For peer review only - http://bmjopen.bmj.com/site/about/guidelines.xhtml

Scirocco
Scaling Integrated Care in Context

and communications, and the power to influence and (sometimes) mandate new working practices. This means alignment of purpose across diverse organisations and professions, and the willingness to collaborate and put the interest of the overall care system above individual incentives. It also means managing the introduction of technology enabled care services in a way that makes them easy to use, reliable, secure, and acceptable to care professionals and citizens alike.

- Enabling properly funded programmes, including a strong programme, project management and change management; establishing ICT or eHealth competence centres to support roll-out; distributed leadership, to reduce dependency on a single heroic leader; excellent communication of goals, progress and successes.
- Managing successful eHealth innovation within a properly funded, multi-year transformation programme.
- Taking into account the need to address the risk of health inequities.
- Establishing organisations with the mandate to select, develop and deliver eHealth services.

Assessment scale:

0 – Fragmented structure and governance in place

1 – Recognition of the need for structural and governance change

2 – Formation of task forces, alliances and other informal ways of collaborating

3 – Governance established at a regional or national level

4 – Roadmap for a change programme defined and accepted by stakeholders involved.

5 – Full, integrated programme established, with funding and a clear mandate.

3. eHealth Services

Objectives:

Integrated care requires, as a foundational capability, sharing of health information and care plans across diverse care teams that lead progressively to systems for enabling continuous collaboration, measuring and managing outcomes, and enabling citizens to take a more active role in their care. This means building on existing eHealth services, connecting them in new ways to support integration, and augmenting them with new capabilities, such as enhanced security and mobility.

- Essential components to enable information-sharing, based on secure and trusted services.
- 'Digital first' policy (where possible, move phone and face-to-face services to digital services to reduce dependence on staff and promote self-service).
- Availability of fundamental building blocks to enable eHealth services ('ICT infrastructure').

For peer review only

For peer review only - http://bmjopen.bmj.com/site/about/guidelines.xhtml

- Data protection and security designed into patient records, registries, online services etc.
- Enabling of new channels for healthcare delivery and new services based on advanced communication and data processing technologies.
- Address the risk of the Digital health divide.

Assessment scale:

0 – There are no eHealth services to support integrated care in place.

1 – There is recognition of need but there is no strategy and/or plan on how to deploy eHealth services to support integrated care.

2 – There is a mandate and plan(s) to deploy regional/national eHealth services across the healthcare system but not yet implemented.

3 – eHealth services to support integrated care are piloted but there is no yet region wide coverage.

4 – eHealth services to support integrated care are deployed widely at large scale.

5 – Universal, at-scale regional/national eHealth services used by all integrated care stakeholders.

4. Standardisation & Simplification

Objectives:

When considering eHealth services and how they can support the information sharing and collaboration needs of integrated care, the task can be made easier if the number of different systems in use, and the formats in which they store data, can be simplified. Practically, this means trying to consolidate data centres, standardising on fewer systems, and agreeing on what technical standards will be used across a region or country.

- Simplification of infrastructure; fewer integration points to manage; easier interoperability and procurement.
- Consolidation of applications and data centres into fewer sites.
- Regional standardisation on fewer (or single) solutions.
- Ability to view and exchange medical data from different systems across diverse care settings.

Assessment scale (too technical language):

0 – No standards in place or planned that support integrated care services

1 – Discussion of the necessity of ICT to support integrated care and of any standards associated with that ICT is initiated

For peer review only - http://bmjopen.bmj.com/site/about/guidelines.xhtml

2 – An ICT infrastructure to support integrated care has been agreed together with a recommended set of technical standards – there may still be local variations or some systems in place are not yet standardised

3 – A recommended set of agreed technical standards at regional/national level; some shared procurements of new systems at regional/national level; some large-scale consolidations of ICT underway

4 – A unified set of agreed standards to be used for system implementations specified in procurement documents; many shared procurements of new systems; consolidated data centres and shared services widely deployed

5 – A unified and mandated set of agreed standards to be used for system implementations fully incorporated into procurement processes; clear strategy for technical specification of new systems in regional/national procurement of new systems; consolidated datacentres and shared services (including the cloud) is normal practice.

5. Funding

Objectives:

Changing systems of care so that they can offer better integration requires initial investment and funding; a degree of operational funding during transition to the new models of care; and on-going financial support until the new services are fully operational and the older ones are de-commissioned. Ensuring that initial and on-going costs can be financed is an essential activity that uses the full range of mechanisms from regional/national budgets to 'stimulus' funds, European Union investment funds, public-private partnerships (PPP) and risk-sharing mechanisms).

Assessment scale:

0 – No additional funding is available to support the move towards integrated care

1 – Funding is available but mainly for the pilot projects and testing

2 – Consolidated innovation funding available through competitions/grants for individual care providers and small-scale implementation

3 – Regional/national (or European) funding or PPP for scaling-up is available

4 – Regional/national funding and/or reimbursement schemes for on-going operations is available

5 – Secure multi-year budget and/or reimbursement schemes, accessible to all stakeholders, to enable further service development.

6. Removal of Inhibitors

Objectives:

For peer review only

For peer review only - http://bmjopen.bmj.com/site/about/guidelines.xhtml

Even with political support, funded programmes and good eHealth infrastructure, many factors can still make integrated care difficult to deliver, by delaying change or limiting how far change can go. These include legal issues with data governance, resistance to change from individuals or professional bodies, cultural barriers to the use of technology, perverse financial incentives, and lack of skills. These factors need to be recognised early, and a plan developed to deal with them, so as to minimise their impact.

- Actions to remove barriers: legal, organisational, financial, skills taking into account the need to address the risk of health inequities.
- Changes to the law concerning e.g., medical acts, information governance, data sharing – factors which may hold up innovation.
- Creation of new organisations or collaborations to encourage cross-boundary working ('normative integration').
- Changes to reimbursement to support behavioural change and process change.
- Education and training to increase understanding of innovations and technology enabled care solutions in order to speed up solution delivery.

Assessment scale:

0 – No awareness of the effects of inhibitors on integrated care

1 – Awareness of inhibitors but no systematic approach to their management is in place

2 – Strategy for removing inhibitors agreed at a high level

3 – Implementation Plan and process for removing inhibitors have started being implemented locally

4 – Solutions for removal of inhibitors developed and commonly used

5 – High completion rate of projects & programmes; inhibitors no longer an issue for service development

7. Population Approach

Objectives:

Integrated care can be developed to benefit those citizens who are not thriving under existing systems of care, in order to help them manage their health and care needs in a better way, and to avoid emergency calls and hospital admissions and reduce hospital stays. This is a practical response to meeting today's demands. Population health goes beyond this, and uses methods to understand where future health risk (and so, demand) will come from. It offers ways to act ahead of time, to predict and anticipate, so that citizens can maintain their health for longer and be less dependent on care services as they age.

For peer review only - http://bmjopen.bmj.com/site/about/guidelines.xhtml

Scirocco
Scaling Integrated Care in Context

- Understanding and anticipating demand; meeting needs better and addressing health inequalities.
- Improving the resilience of care systems by using existing data on public health, health risks, and service utilisation.
- Taking steps to divert citizens into more appropriate and convenient care pathways based on user preferences.
- Predicting future demand and taking steps to reduce health risks though technology-enabled public health interventions.

Assessment scale:

0 – Population health approach is not applied to the provision of integrated care services

1 – Population-wide risk stratification considered but not started

2 – Risk stratification approach is used in certain projects on an experimental basis

3 – Risk stratification used for specific groups i.e. those who are at risk of becoming frequent service users –

4 – A population risk approach is applied to integrated care services but not yet systematically or to the full population

5 – Whole population stratification deployed and fully implemented.

8. Citizen Empowerment

Objectives:

Health and social care systems are under increasing pressure to respond to demands that could otherwise be handled by citizens and carers themselves. The evidence suggests that many individuals would be willing to do more to participate in their own care if easy-to-use services, such as appointment booking, self-monitoring of health status, and alternatives to medical appointments, were available to them. This means providing services and tools that enable convenience, offer choice, and encourage self-service and engagement in health management, taking into account the need to address the risk of health inequities.

Assessment scale:

0 – Citizen empowerment is not considered as part of integrated care provision

1 – Citizen empowerment is recognised as important part of integrated care provision but effective policies to support citizen empowerment are still in development

For peer review only

For peer review only - http://bmjopen.bmj.com/site/about/guidelines.xhtml

2 - Citizen empowerment is recognised as important part of integrated care provision, effective policies to support citizen empowerment are in place but citizens do not have access to health information and health data

3 - Citizens are consulted on integrated care services and have access to health information and health data

4 – Incentives and tools exist to motivate and support citizens to co-create healthcare services and use these services to participate in decision-making process about their own health

5 – Citizens are fully enaged in decision-making processes about their health, and are included in decsion-making on service delivery and policy-making.

9. Evaluation Methods

Objectives:

As new care pathways and services are introduced to support integrated care, there is a clear need to ensure that the changes are having the desired effect on quality of care, cost of care, access and citizen experience. This supports the concept of evidence-based investment, where the impact of each change is evaluated, e.g. by health economists working in universities or in special agencies. Health technology assessment (HTA) is an important method here, and can be used to justify the cost of scaling up of integrated care to regional or national level.

- Establishing baselines (on cost, quality, access etc.) in advance of new service introduction.
- Systematically measuring the impact of new services and pathways using appropriate methods (e.g., observational studies, incremental improvement, clinical trials).
- Generating evidence that leads to faster adoption of good practice.

Assessment scale:

0 – No evaluation of integrated care services is in place or in development

1 – Evaluation of integrated care services is planned to take place and be established as part of a systematic approach

2 – Evaluation of integrated care services exists, but not as a part of a systematic approach

3 – Some integrated care initiatives and services are evaluated as part of a systematic approach

4 – Most integrated care initiatives are subject to a systematic approach to evaluation; published results

5 – A systematic approach to evaluation, responsiveness to the evaluation outcomes, and evaluation of the desired impact on service redesign (i.e., a closed loop process).

For peer review only - http://bmjopen.bmj.com/site/about/guidelines.xhtml

10. Breadth of Ambition

Objectives:

Integrated care includes many levels of integration, such as integration between primary and secondary care, of all stakeholders involved in the care process, or across many organisations. It may be developed simply for healthcare needs (i.e., vertical integration) or it may include social workers, the voluntary sector, and informal care (i.e., horizontal integration). The broader the ambition, the more numerous and diverse the stakeholders who have to be engaged. Similarly, integration may include all levels of the system or may be limited to clinical information sharing. The long-term goal should be fully integrated care services which provide a complete set of seamless interactions for the citizen, leading to better care and improved outcomes.

- Integration supported at all levels within the healthcare system – at the macro (policy, structure), meso (organisational, professional) and micro (clinical) levels.
- Integration between the healthcare system and other care services (including social, voluntary, informal, family services).
- Seamless transition for the patient between and within care services.

Assessment scale:

0 – Coordination activities arise but not as a result of planning or the implementation of a strategy

1 – The citizen or their family may need to act as the integrator of service in an unpredictable way

2 – Integration within the same level of care (e.g., primary care) is achieved

3 – Integration between care levels (e.g., between primary and secondary care) is achieved

4 – Improved coordination of social care service and health care service needs is introduced

5 – Fully integrated health & social care services are in place and functional.

11. Innovation Management

Objectives:

Many of the best ideas are likely to come from clinicians, nurses and social workers who understand where improvements can be made to existing processes. These innovations need to be recognised, assessed and, where possible, scaled up to provide benefit across the system. At the same time, universities and private sector companies are increasingly willing to engage in open innovation, and innovative procurement, in order to develop new technologies, test process improvements and deliver new services that meet the needs of citizens. There is also value in looking outside the system to other regions and countries that are dealing with the same set of challenges, to learn from their experiences. Overall, this means managing the innovation process to get the best results for the systems of care, and ensuring that good ideas are encouraged and rewarded.

For peer review only

For peer review only - http://bmjopen.bmj.com/site/about/guidelines.xhtml

- Adopting proven ideas faster.
- Enabling an atmosphere of innovation from top to bottom, with collection and diffusion of best practice.
- Learning from inside the system, as well as from other regions, to expand thinking and speed up change.
- Involving regional health and social care authorities, universities and private sector companies and other sectors in the innovation process (i.e., ‘open innovation’).
- Using innovative procurement approaches (Pre-Commercial Procurement, IPP, PPP, Shared Risk, Outcome-Based Payment)
- Using European projects and partnerships (e.g., Horizon 2020, EIP, CEF, ERDF, ESIF).

Assessment scale:

0 – No innovation management in place

1 – Innovation is encouraged but there is no overall plan

2 – Innovations are captured and there are some mechanisms in place to encourage knowledge transfer

3 – Formalised innovation management process is planned and partially implemented

4 – Formalised innovation management process is in place and widely implemented

5 – Extensive open innovation combined with supporting procurement & the diffusion of good practice is in place

12. Capacity Building

Objectives:
Capacity building is the process by which individual and organisations obtain, improve and retain the skills and knowledge needed to do their jobs competently. As the systems of care are transformed, many new roles will need to be created and new skills developed. These will range from technological expertise and project management, to successful change management. The systems of care need to become ‘learning systems’ that are constantly striving to improve quality, cost and access. They must build their capacity so as to become more adaptable and resilient. As demands continue to change, skills, talent and experience must be retained. This means ensuring that knowledge is captured and used to improve the next set of projects, leading to greater productivity and increasing success.

- Increasing skills; continuous improvement.

For peer review only - http://bmjopen.bmj.com/site/about/guidelines.xhtml

- Building a skill base that can bridge the gap and ensure that the capacity needs are understood and addressed by ICT where appropriate
- Providing tools, processes and platforms to allow organisations to assess themselves and build their own capacity to deliver successful change.
- Creating an environment where service improvements are continuously evaluated and delivered for the benefit of the entire care system.

Assessment scale:

0 – Integrated care services are not considered for capacity building

1 – Some approaches to capacity building for integrated care services are in place

2 – Cooperation on capacity building for integrated care is growing across the region

3 – Learning about integrated care and change management is in place but not widely implemented

4 – Systematic learning about integrated care and change management is widely implemented; knowledge is shared, skills retained and there is a lower turnover of experienced staff

5 – A 'person-centred learning healthcare system' involving reflection and continuous improvement.

For peer review only - http://bmjopen.bmj.com/site/about/guidelines.xhtml

Appendix B Details of the interviews

Participants	Main theme discussed	Time of interview
Project members responsible for the coordination of the project (2 members)	Views and experiences in undertaking the work regarding the coordination of the project.	August 2018
Project members responsible for implementing the first step of the strategy (3 members)	Views and experiences in undertaking the work regarding identification of maturity requirements of selected local IC interventions (GPs) which have the potential for scaling up	First interview in June 2017; second interview on revised methodology September 2018
Project members responsible for the developments of the tool within the strategy (2 members)	Views and experiences in undertaking the work on the refinement of MM	August 2018
Project members responsible for the implementation of the second step in the strategy (2 members)	Views and experiences in undertaking the work regarding the self-assessment process of SCIROCCO regions	July 2018
Project members responsible for the implementation of the third step in the strategy (2 members)	Views and experiences in undertaking the work regarding coaching and twinning of regions	July 2018
Project members responsible for the collecting lessons learned on using the tool and the process of scaling up (1 member, and 1 member provided feedback to the summary of interview)	Views and experiences in undertaking the work regarding the collection lessons learned on the process of scaling up	July 2018

For peer review only

For peer review only

Appendix C Timetable planning project and executed activities

Blue: anticipated duration of task. Orange: actual duration of task spent during implementation as indicated in reports. Yellow: deviated actual duration spent as indicated by other reports.

	1									2												3											4
Month	Apr 16 (M1)	May 16 (M2)	June 16 (M3)	July 16 (M4)	August 16(M5)	Sep 16 (M6)	Oct 16 (M7)	Nov 16 (M8)	Dec 16 (M9)	Jan 17 (M10)	Feb 17 (M11)	Mar 17 (M12)	Apr 17 (M13)	May 17 (M14)	June 17 (M15)	July 17 (M16)	Aug 17 (M17)	Sep 17 (M18)	Oct 17 (M19)	Nov 17 (M20)	Dec 17 (M21)	Jan 18 (M22)	Feb 18(M23)	Mar 18 (M24)	Apr 18 (M25)	May 18 (M26)	June 18 (M27)	July 18 (M28)	Aug 18 (M29)	Sep 18 (M30)	Oct 18 (M31)	Nov 18 (M32)	
Step 1 Maturity requirements in GPs in five regions																																	
Viability assessment of GP																																	
Data collection																																	
Maturity requirements																																	
							1st																2nd										
Step 2 Self-assessment process in five regions																																	
Self-assessment process in five European regions																																	
Strengths and weaknesses of the European regions in integrated care																																	

Step 3 Twinning and coaching activities in five regions																																	
Development of methodology for twinning and coaching																																	
Coaching and twinning acitvities																																	
Development of Action Plans																																	

er review only

COREQ (COnsolidated criteria for REporting Qualitative research) Checklist

.

Topic	Item No.	Guide Questions/Description	Reported on Page No.
**Domain 1: Research team and reflexivity**			
Personal characteristics			
Interviewer/facilitator	1	Which author/s conducted the interview or focus group?	8
Credentials	2	What were the researcher's credentials? E.g. PhD, MD	8
Occupation	3	What was their occupation at the time of the study?	8
Gender	4	Was the researcher male or female?	8
Experience and training	5	What experience or training did the researcher have?	8
Relationship with participants			
Relationship established	6	Was a relationship established prior to study commencement?	8
Participant knowledge of the interviewer	7	What did the participants know about the researcher? e.g. personal goals, reasons for doing the research	8
Interviewer characteristics	8	What characteristics were reported about the inter viewer/facilitator? e.g. Bias, assumptions, reasons and interests in the research topic	19
**Domain 2: Study design**			
Theoretical framework			
Methodological orientation and Theory	9	What methodological orientation was stated to underpin the study? e.g. grounded theory, discourse analysis, ethnography, phenomenology, content analysis	9
Participant selection			
Sampling	10	How were participants selected? e.g. purposive, convenience, consecutive, snowball	8
Method of approach	11	How were participants approached? e.g. face-to-face, telephone, mail, email	15
Sample size	12	How many participants were in the study?	8 and 9
Non-participation	13	How many people refused to participate or dropped out? Reasons?	19
Setting			
Setting of data collection	14	Where was the data collected? e.g. home, clinic, workplace	8
Presence of non-participants	15	Was anyone else present besides the participants and researchers?	8
Description of sample	16	What are the important characteristics of the sample? e.g. demographic data, date	8 and 9
Data collection			
Interview guide	17	Were questions, prompts, guides provided by the authors? Was it pilot tested?	8
Repeat interviews	18	Were repeat inter views carried out? If yes, how many?	NA
Audio/visual recording	19	Did the research use audio or visual recording to collect the data?	8
Field notes	20	Were field notes made during and/or after the inter view or focus group?	8
Duration	21	What was the duration of the inter views or focus group?	8
Data saturation	22	Was data saturation discussed?	9
Transcripts returned	23	Were transcripts returned to participants for comment and/or	9

Topic	Item No.	Guide Questions/Description	Reported on Page No.
		correction?	
**Domain 3: analysis and findings**			
Data analysis			
Number of data coders	24	How many data coders coded the data?	9
Description of the coding tree	25	Did authors provide a description of the coding tree?	9
Derivation of themes	26	Were themes identified in advance or derived from the data?	9
Software	27	What software, if applicable, was used to manage the data?	9
Participant checking	28	Did participants provide feedback on the findings?	NA
Reporting			
Quotations presented	29	Were participant quotations presented to illustrate the themes/findings? Was each quotation identified? e.g. participant number	15 and 16
Data and findings consistent	30	Was there consistency between the data presented and the findings?	9
Clarity of major themes	31	Were major themes clearly presented in the findings?	9-18
Clarity of minor themes	32	Is there a description of diverse cases or discussion of minor themes?	18

Developed from: Tong A, Sainsbury P, Craig J. Consolidated criteria for reporting qualitative research (COREQ): a 32-item checklist for interviews and focus groups. *International Journal for Quality in Health Care*. 2007. Volume 19, Number 6: pp. 349 – 357

For peer review only
